# Characteristic Aroma Fingerprint Disclosure of Apples (*Malus* × *domestica*) by Applying SBSE-GC-O-MS and GC-IMS Technology Coupled with Sensory Molecular Science

**DOI:** 10.3390/foods15030482

**Published:** 2026-01-30

**Authors:** Ning Ma, Jiancai Zhu, Heng Wang, Michael C. Qian, Zuobing Xiao

**Affiliations:** 1 Department of Food Science & Technology, School of Agriculture & Biology, Shanghai Jiao Tong University, Shanghai 200240, China; 2 School of Perfume and Aroma Technology, Shanghai Institute of Technology, Shanghai 201418, China; 3 Department of Food Science and Technology, Oregon State University, Corvallis, OR 97331, USA

**Keywords:** aroma-active compounds, HS-GC-IMS, SBSE, aroma recombination, PCA, PLSR

## Abstract

Apple aroma is an important factor influencing consumers’ preferences. To understand the overall flavor characteristics of apples (Ruixue, Liangzhi, Grystal Fuji, and Guifei), volatile compounds and aroma profiles were investigated by headspace–gas chromatography–ion mobility spectrometry (HS-GC-IMS) combined with stir bar sorptive extraction (SBSE) and gas chromatography–mass spectrometry (GC-MS). The results showed that a total of 56 aroma compounds were identified by SBSE-GC-MS, and 39 aroma-active compounds were screened out using aroma intensity (AI) and odor activity value (OAV). Aroma recombination experiments showed enhanced ‘fruity’ and ‘sweet’ notes, whereas ‘floral’, ‘woody’, and ‘green’ aromas were weaker compared to the Crystal Fuji sample. Additionally, GC-IMS coupled with principal component analysis (PCA) was used to distinguish the apple samples, and partial least squares regression (PLSR) was applied to explore the correlation between sensory attributes and characteristic aroma compounds. The results indicated that Crystal Fuji exhibited the greatest correlation with the “woody” attribute, and Ruixue was highly correlated with “fruity”, “green”, and “sour” attributes, while butanoic acid, β-damascenone, butyl acetate, pentyl acetate, furfuryl alcohol, γ-decalactone, and vanillin had a significant impact on the “flower” and “sweet” attributes of Guifei. This study clarified the characteristic aroma composition of the four apple cultivars, providing data support for apple flavor quality evaluation and cultivar optimization.

## 1. Introduction

Aroma is an important quality attribute of various fruits and beverages, including apples [1,2,3]. Apples are attracting widespread attention from both consumers and researchers due to their unique flavor characteristics. Apples contain a wide variety of volatile compounds, but only a limited proportion of these compounds can be olfactorily detectable by consumers [4]. The characteristic flavor of different apple varieties is different, and they are often distinguished based on the types and contents of volatile compounds according to the variety, origin, and maturity of apples [5]. For example, Li et al. identified 130 volatile compounds from “Qin Cui” and “Qin Mi” by headspace solid-phase microextraction combined with comprehensive two-dimensional gas chromatography–quadrupole time-of-flight mass spectrometry (SPME-GC × GC-QTOFMS), among which 5 compounds were screened as the key differential compounds for distinguishing apple varieties [6]. In recent years, many researchers found that the aroma profile of apples varies at different growth stages, and the maturity of apples could be judged based on their aroma [4,7]. Zhao et al. [8] comparatively studied the key aroma components of red-fleshed apples in Xinjiang, from the young fruit period through the expansion period and the conversion period to the ripening period. They found that the concentration of bound-form aroma compounds was higher than that of free-form compounds throughout the four periods, and total aromas were found to have the lowest concentration in the expansion period and the highest concentration in the ripening period. Li et al. [9] examined the accumulation of aroma volatiles and unsaturated fatty acids, alongside the expression of fatty acid metabolism-related genes, in apple fruit stored under low-oxygen conditions. They found that low-oxygen storage impaired fatty acid metabolism by repressing ethylene-mediated expression of MdERF74 and MdERF75, ultimately hindering aroma production in apple fruit.

Volatile compounds in apples are mostly identified by GC-MS [5,8,10]. Currently, there are also a few research reports that GC × GC-QMS or GC × GC-QTOFMS were used to analyze volatile compounds in apples [3,4,6]. Recently, among the methods for identifying food aroma compounds, we have found that GC-IMS is also a good method for detecting volatile compounds. This technique enables rapid qualitative analysis of volatile components with high sensitivity to low-molecular-weight compounds. Moreover, it combines the advantage of gas chromatography in volatile separation and the advantages of ion mobility spectroscopy in identifying each compound based on the difference in ion migration rates in an electric field [11]. GC-IMS has been widely used in the analysis of volatile compounds in tea, fruits, wine, etc. [12,13,14]. In addition, chemometrics techniques (such as PCA and PLSR) could be combined with GC-IMS to offer helpful strategies for food classification. Nevertheless, a noticeable limitation of HS-GC-IMS technology is that there is no complete database, and it cannot be easily used for accurate quantitative analysis due to its nonlinear response [15]. Therefore, the analysis could be conducted simultaneously by GC-IMS and GC-MS. On the one hand, it enables accurate quantitative analysis of aroma compounds in apples; on the other hand, it can quickly distinguish differences between samples. This complementary combination addresses the limitations of single-platform analysis, ensuring the comprehensive and reliable characterization of apple aroma profiles. This analysis provides a new idea for the more accurate analysis and identification of volatile compounds in various foods.

It is well known that the volatile compounds of apples are quite complicated and are found at trace levels [4,8,10]. In particular, the composition and content of volatile compounds varies greatly when various extraction methods are employed. Thus, the selection of an appropriate extraction method has become a prerequisite to enhance the understanding of apple volatiles. In general, the volatile enrichment technique most often used in apples is HS-SPME, and solvent-assisted flavor evaporation (SAFE) has also been applied in the extraction of apple aroma compounds in recent years. However, HS-SPME has limitations such as limited adsorption capacity and competitive adsorption in complex matrices. Although SAFE can extract high-boiling-point volatile compounds, its operation is rather complex and time-consuming, and it carries a high risk of solvent residue. In contrast, SBSE has a very high enrichment for multiple volatile compounds, good extraction efficiency and reproducibility, simple operation, and wide application range [16,17]. At present, it has not been widely applied in the extraction of apple aroma. Therefore, in this experiment, we utilized the advantages of SBSE, which enhances the enrichment efficiency of apple aroma compounds, shortens extraction time, and addresses the gap in existing extraction methods for apple aroma compounds.

Furthermore, OAV is an important indicator for measuring the contribution of volatile compounds to the characteristic flavor of food; it is often used to assess the importance of aroma compounds to the overall aroma profile [2,4,18]. OAV is not only related to the concentration of aroma compounds, but also to its threshold. However, the odor thresholds previously used to calculate OAVs were often based on values reported in aqueous solutions [10,19,20]. In reality, the aroma we perceive originates from the actual apple matrix, rather than water. Thus, measuring the odor thresholds of aroma compounds directly in the apple matrix will improve the accuracy of OAV calculations, thereby enabling more precise screening of the key aroma compounds that contribute significantly to apple flavor.

Therefore, in this study, HS-GC-IMS was used to analyze the characteristic volatile compounds in four apple samples, allowing rapid differentiation of apple varieties. Aroma compounds in apples were then qualitatively and quantitatively analyzed using SBSE coupled with GC-MS. This approach improved the enrichment efficiency of volatile compounds and enabled the detection of a more comprehensive range of aroma compounds. The aroma-active compounds in apples were identified by the AIs coupled with gas chromatography–olfactometry (GC-O) analyses and the OAVs. Moreover, the olfactory thresholds of aroma-active compounds were detected by three-alternative forced-choice presentation (3-AFC). Finally, aroma recombination experiments confirmed that the screened aroma-active compounds were the key components contributing to the characteristic flavor of apples. This research will provide a scientific basis for further differentiating apple types and the selection of new superior apple varieties based on flavor quality guidance.

## 2. Materials and Methods

### 2.1. Chemicals

Ethyl acetate (99.8%), propyl acetate (≥99.5%), 2-methylpropyl acetate (99%), pentyl acetate (99%), 1-butanol (99.9%), 2-methyl-1-butanol (99%), hexyl hexanoate (≥98%), ethyl heptanoate (99%), 2-methylbutyl acetate (99%), hexyl 2-methylbutanoate (≥98%), phenylacetaldehyde (≥95%), (E,E)-2,4-hexadienal (≥95%), 2-octanone (99%), 6-methyl-5-hepten-2-one (99%), β-damascenone (97%), acetic acid (≥99.5%), propanoic acid (≥98%), butanoic acid (99%), hexanoic acid (≥99%), benzoic acid (≥99%), vanillin (99%), and D-limonene (≥99%) were purchased from Sigma-Aldrich, Shanghai, China. Butyl acetate (99%), trans-2-hexenal (97%), butyl propanoate (99%), hexyl butanoate (98%), and 2-ethylhexanol (99.5%) were purchased from TCI (Shanghai) Development Co., Ltd. (Shanghai, China). Hexyl acetate (99%), octyl acetate (99%), benzyl acetate (≥99%), phenethyl acetate (99%), 2-ethylhexyl acetate (99%), methyl butanoate (≥99%), ethyl butanoate (99%), butyl butanoate (99%), 1-hexanol (≥99%), 2-heptanol (≥99%), acetaldehyde (≥99.5%), butanal (≥99.5%), 3-methylbutanal (≥98%), hexanal (98%), benzaldehyde (99.5%), 6-methyl-5-hepten-2-ol (98%), linalool (96%), citronellol (≥95%), benzyl alcohol (≥99%), phenethyl alcohol (≥99.5%), α-terpineol (≥98%), 3-methylthiopropanol (98%), γ-hexalactone (≥98%), γ-octalactone (≥99%), γ-decalactone (≥98%), γ-undecalactone (≥98%), 5-methyl furfural (≥98%), furfuryl alcohol (99%), and furfural (99%) were purchased from Adamas-beta (Shanghai) Co., Ltd. (Shanghai, China). 2-Methylpropyl butanoate (≥98%) was purchased from Aladdin Reagent (Shanghai) Co., Ltd. (Shanghai, China). d-Glucose, d-fructose, sucrose, sorbitol, malic acid, citric acid, and tartaric acid were purchased from Adamas-beta (Shanghai) Co., Ltd. (Shanghai, China). The above standard compounds and experimental reagents were analytical grade. The deionized water used in the experiments was obtained from the Milli-Q purification system. A series of C7–C30 n-alkane standards was obtained from Titan Technology (Shanghai, China) with the number 041295896.

### 2.2. Materials

Four apple cultivars (Ruixue, Crystal Fuji, Liangzhi, and Guifei) were carefully selected from Baishui and Luochuan counties in Shaanxi Province, Haiyang city in Shandong Province, and Qingyang city in Gansu Province, respectively. Mature apples were immediately transported to the laboratory using a cold chain and stored at 4 °C. Apples free of physical and mechanical damage had their peel and seeds quickly removed, then their flesh was cut into 2 cm pieces and frozen in liquid nitrogen, put into a zip-lock bag, and stored at −80 °C in a refrigerator for the subsequent experiments [3]. During the experiment, we took apple samples out of the refrigerator, allowed them to reach room temperature, and then put them into a blender to prepare the juice.

### 2.3. Analysis of Volatile Compounds by HS-GC-IMS

The identification of apple samples was achieved by the HS-GC-IMS instrument as reported previously [21], which was a combined device of an Agilent 490 gas chromatograph (Agilent Technologies, Palo Alto, CA, USA) equipped with a MXT-WAX capillary column (30 m × 0.53 mm, 1 μm, Restek, Bellefonte, PA, USA) and IMS instrument (FlavourSpec^®^, Gesell-schaft für Analytische Sensorsysteme mbH, Dortmund, Germany), fitted with an automated headspace sampler unit (Solid Phase Micro Extraction, 57330- U, Supelco, Bellefonte, PA, USA).

The apple samples were placed into a 20 mL headspace (HS) vial, and then incubated at 40 °C at a rotation speed of 500 rpm for 20 min. After incubation, 500 µL of headspace gas was automatically injected under the splitless injection mode, and the injector temperature was kept at 85 °C. Subsequently, the samples were transferred into the MXT-WAX capillary column by nitrogen (99.99%) at a programmed flow as follows: initially 2 mL/min for 2 min, then increased to 10 mL/min for 10 min, after which the flow was ramped to 100 mL/min for 10 min, and eventually increased to 150 mL/min for 5 min. The analytes were separated at 60 °C in the column and then transferred to the 45 °C IMS ionization chamber to be ionized. The resulting ions were directed to the drift tube (9.8 cm in length) under the drift gas (ultrapure nitrogen) of 150 mL/min and programmed at a constant temperature (45 °C). The volatile compounds were tentatively identified by comparing the retention index (RI) and drift time with a combination of Laboratory Analytical Viewer software (VOCal 0.4.x) and the NIST 14 database of DB-5/HP-5 in the GC-IMS Library Search software (Version 1.0.3, G.A.S., Dortmund, Germany). Furthermore, the relative concentration of the volatile components was estimated according to the peak intensity in HS-GC-IMS. The RI values of volatile compounds were calculated using the C4–C9 n-Ketones (Sinopharm Chemical Reagent Beijing Co., Ltd., Beijing, China) as external standards. The chromatographic conditions were the same as the samples. The experiment was repeated three times.

### 2.4. Volatile Compounds Extraction by SBSE

A PDMS (polydimethylsiloxane)-coated stirring bar (10 mm length, 0.5 mm thickness, 24 μL capacity) was selected to extract volatile compounds in four apple samples. This PDMS bar was purchased from Gerstel (Mülheim an der Ruhr, Germany). The extraction conditions were optimized: extraction time, temperature, and sample content were 45 min, 45 °C, and 8 g, respectively. The SBSE bar was immersed into 8 g of apple juice sample with 10 μL of 2-octanol (400 mg/L) as an internal standard, and then magnetically stirred at 600 rpm at a constant temperature of 45 °C. After extraction for 45 min, the SBSE bar was washed with a small amount of distilled water, dried with lint-free paper, and then transferred to a thermal desorption unit for GC-MS analysis. The thermal desorption systems (TDS) and the cooling injector system (CIS) conditions have been slightly modified based on previous studies [18,22]. The temperature program of the TDS was maintained at 40 °C for 6 min and then increased to 230 °C for 12 min at 40 °C/min under the splitless mode. Then, the analyte was focused in the CIS at a low temperature of −70 °C. The procedure temperature of the CIS increased from −70 °C to 240 °C at a rate of 10 °C/s. The experiment was repeated three times.

### 2.5. GC-O Analysis

The GC separation consisted of an Agilent 7890 chromatograph equipped with an olfactory detector port (ODP-3, Gerstel, Mulheim an der Ruhr, Germany). This system allowed us to obtain the odor characteristics of each compound detected by the sniffing port. The GC effluent was split 1:1. The compounds were separated on DB-5 and HP-Innowax columns (60 m × 0.25 mm × 0.25 μm, Agilent, Santa Clara, CA, USA). The initial temperature of the oven was 40 °C, which was kept for 6 min, ramped to 100 °C at a rate of 3 °C/min and held for 2 min, and finally, ramped at a rate of 5 °C/min to 230 °C for 20 min. Moist air was pumped into the sniffing port at a flow rate of 50 mL/min to quickly remove the residual odorant eluted from the sniffing port and to relieve the discomfort of the panelist from dehydration of the nasal mucosa. The aroma-active compounds were perceived by 12 professional sensory panelists (6 females and 6 males, aged from 22 to 33), who were healthy, non-smoking regular members with no odor/taste disorders. They were selected based on the protocol for human subject study approved by the school’s Human Ethics Committee (B20250330I). Prior to sensory analysis, all panelists underwent training to familiarize themselves with odor descriptors using solutions of artificial odorants. During the evaluation, the aroma-active compounds detected by panelists were recorded, including the onset and cessation times during effluent sampling via the sniffing mask [3]. They recorded the start and end time, odor description, and AI while sniffing the effluent from the sniffing mask. The AI was evaluated using the 10-point intensity scale from 0 to 10, where “0” represented none, “5” was moderate intensity, and “10” was extreme intensity. The experiment was replicated in triplicate by each panelist. Finally, the final AI score of each effluent was the average score of 12 sensory panelists. The other detailed GC-O analysis referred to our previous study [3].

### 2.6. Qualitative and Quantitative Analysis of Aroma Compounds in Apple Samples by GC-MS

The a GC-MS (Agilent, Santa Clara, CA, USA) instrument was used to separate and identify volatile compounds. Two chromatographic columns were used for sample analysis: a 60 m × 0.25 mm × 0.25 μm HP-Innowax polar column and a 60 m × 0.25 mm × 0.25 μm DB-5 non-polar column. The instrumental conditions for analyzing odorants are as follows. The carrier gas (helium) flow rate was maintained at 2 mL/min throughout the analysis. The ion energy for electron impact was 70 eV. The injector, ion source, and quadruple mass filter temperatures were set to 250 °C, 230 °C, and 150 °C, respectively. The initial temperature of the oven was kept at 40 °C for 5 min, then ramped to 100 °C at a rate of 3 °C/min and held for 2 min, and finally, raised at a rate of 5 °C/min to 230 °C for 20 min. Samples were injected in the splitless mode. The ion-scanning range was set to vary between 30 and 450. The Retention Index (RI) is a widely applied parameter in chromatographic analysis, which characterizes the retention behavior of compounds. The RIs of compounds were calculated based on the retention time of C7–C30 n-alkane standards under the same chromatographic conditions as apple samples. Aroma compounds were identified by matching their RIs, authentic standards, and mass spectra in the NIST 17 and Wiley7n.L databases. All experiments were repeated three times.

The standard curves were established in an apple matrix solution, and the quantitative analysis of aroma compounds was realized. The apple matrix solution was prepared by 55.24 g/L fructose, 32.07 g/L sucrose, 18.53 g/L glucose, 6.07 g/L sorbitol, 2.81 g/L malic acid, 0.05 g/L citric acid, and 0.04 g/L tartaric acid in Milli-Q deionized water [23]. The standards of all volatile compounds identified by SBSE in the four apple cultivars were combined in the model matrix to make up the recombination. The reconstitution was diluted in a gradient of concentrations (1:2, 1:5, 1:10, 1:20, 1:50, and 1:100) with apple matrix solution, and then the recombinant solutions were extracted by SBSE technique, which was held consistent with the procedure for detecting apple samples.

The aroma compounds were quantitatively analyzed by selective ion monitoring (SIM) mass spectrometry. The quantitation ions were carefully selected to ensure the lowest interference and highest response [1]. The external standard curves of aroma compounds are established in which y represents the ratio of the peak area (volatile standard compound peak area/internal standard compound peak area), and x represents the ratio of concentration (the concentration of the volatile standard/the internal standard compound concentration). The quantitation process was repeated three times, the average of which was considered the concentration of the volatiles.

### 2.7. Detection of Odor Thresholds in Apple Model Solution

The olfactory thresholds of 56 aroma compounds were determined by a 3-AFC method and measured in the apple model solution [10]. Both the aroma characteristics and intensities of the compounds were assessed to improve sensory accuracy. Thirty professional sensory panelists (13 females and 17 males, aged from 22 to 35) were selected based on the protocol for human subject study approved by the school’s Human Ethics Committee, and all the panelists were physically healthy, non-smoking regular members with no odor/taste disorders. The sensory group received training for 50 h, 2 h a day, to familiarize and evaluate the aroma compounds of apples. The panelists conducted 10 forced-choice tests for each aroma compound in the formal experiment, with two as the dilution factor, starting from the highest concentration. Only one of the three vials contained aromatic compounds; the other two contained blank controls. If the panelists could identify the sample containing the aroma compound out of three samples, the next sample with a lower concentration would be tested, and so on, until it could not be correctly identified. Finally, the threshold was calculated according to a published formula [10,24], and the corrected detection probability was calculated using a correction formula (1). The sigmoid curve in Equation (2) was used to fit the concentrations and detection threshold. The abscissa corresponding to the ordinate P = 0.5 was the experimental threshold value of the mixture. All experiments were repeated three times.

(1) $$P=\frac{3p-1}{2}$$

where P is the correction value of the detection probability and p is the actual measured detection probability value.

(2) $$P=\frac{1}{1+e^{\left(-\frac{x-x0}{D}\right)}}$$

where x represents the concentration of the odorant [log (μg/kg)], x0 is the olfactory threshold of the odorant [log (μg/kg)], D is the parameter characterizing each odorant, which defined the slope of the function, and P represents the probability of detection (after correction).

### 2.8. OAV

OAV was defined as the ratio of the concentration of a single aroma compound to its corresponding odor threshold [25]. The OAV of quantitative compounds was used to evaluate the contribution of aroma compounds in apples. The threshold values were usually obtained from the published studies, especially the threshold that was detected in water. However, the olfactory thresholds of the aroma compounds were determined in this study. Aroma compounds with OAV ≥ 1 were generally considered to contribute significantly to the aroma characteristics of each sample, although there are a few exceptions where odorants with high OAVs are suppressed in the aroma and compounds with lower OAVs are important contributors [26].

### 2.9. Aroma Recombination

It is well known that the overall aroma of a mixture composed of aroma compounds is unpredictable. Therefore, in order to confirm that volatile compounds with high OAVs are important volatiles for apples, aroma recombination was carried out. The apple model matrix (55.24 g/kg fructose, 32.07 g/kg sucrose, 18.53 g/kg glucose, 6.07 g/kg of sorbitol, 2.81 g/kg of malic acid, 0.05 g/kg of citric acid, and 0.04 g/kg of tartaric acid) was applied as the matrix for the recombination model. Then, the AR was constructed based on the selected aroma-active compounds (OAVs ≥ 1) detected in Crystal Fuji apple with strict reference to the natural concentration in the fruit. Artificial sensory evaluation was used to prove the aroma recombination experiment, and 12 panelists who participated in the GC-O-MS experiment were asked to evaluate the aroma intensity of each attribute of the recombination model on a scale of 0 to 10. The procedure can be found in more detail in previous studies [3,27].

### 2.10. Statistical Analysis

The statistical analysis was displayed as mean ± standard deviation. SPSS version 26.0 (SPSS Inc., Chicago, IL, USA) was used to conduct a one-way analysis of variance. Significant differences (*p* < 0.05) in the content of volatile aroma compounds among samples were analyzed by Duncan’s multiple comparison test. The GC-IMS data was analyzed by the Reporter plug-in and Gallery Plot plug-in in LAV (G.A.S., Dortmund, Germany). PLSR was conducted using the Unscrambler X 10.4 (CAMO ASA, Oslo, Norway) to perform the correlation analysis on apple samples, sensory attributes, and aroma-active compounds.

## 3. Results

### 3.1. Aroma-Active Volatiles Recognized by GC-O

As shown in Table 1, a total of 24, 32, 41, and 39 aroma compounds were identified in Ruixue, Liangzhi, Crystal Fuji, and Guifei, respectively, and further analysis showed that Crystal Fuji had the richest aroma composition, which may be closely related to its unique flavor characteristics. According to the olfactory results, butyl acetate (AI: 5.2 to 8.8), hexyl acetate (AI: 7.3 to 8.1), 2-methylbutyl acetate (AI: 6.5 to 8.8), (E)-2-hexenal (AI: 6.5 to 7.6), 1-hexanal (AI: 5.5–7.2), pentyl acetate (AI: 6.1 to 6.9), butyl butanoate (AI: 5.3 to 6.1), 1-hexanol (AI: 5.1 to 6.3), and so on, were the most powerful aroma-active compounds which show strong aroma intensities, and those compounds played an important role in the unique aroma of apples.

Esters were an important factor in the richness of apple flavor [28,29], which played a key role in the “fruity, sweet” notes of apples [8,10,27,30]. Butyl acetate, hexyl acetate, 2-methylbutyl acetate, pentyl acetate, ethyl butanoate, 2-methylbutyl butanoate, and so on presented relatively high AIs. Previous studies disclosed that acetates (especially butyl acetate, 2-methylpropyl acetate, 2-methylbutyl acetate, and hexyl acetate), butanoates (methyl butanoate, ethyl butanoate, and butyl butanoate), and hexanoates (hexyl hexanoate) played important roles in the aroma of ripe apples [27,31], which is consistent with the results of this study.

Butanal (AI: 3.7 to 3.8), hexanal (AI: 5.5 to 7.2), (E)-2-hexenal (AI: 6.5 to 7.6), and (E,E)-2,4-hexadienal (AI: 3.5–3.7) were identified at relatively high AIs, contributing to the “green, grass” flavor of the apples. Previous studies have found that C6 aldehydes and C6-alcohols mainly contribute green, grassy, and vegetable notes to apple aroma [32]. GC-O analysis revealed that alcoholic compounds contributed “green, fresh, fruity” sensory notes. Butanol (AI: 5.9 to 6.8), hexanol (AI: 5.1 to 6.3), 6-methyl-5-heptene-2-ol (AI: 2.8 to 3.7), 2-heptanol (AI: 3.8), linalool (AI: 4.5 to 4.7), phenethyl alcohol (AI: 3.5 to 3.8), 3-methylthiopropanol (AI: 3.3 to 3.9), 2-methyl-1-butanol (AI: 4.1 to 6.2), α-terpineol (AI: 3.8), citronellol (AI: 3.8), and 2-ethylhexanol (AI: 1.9 to 2.3) were detected in four apple samples from Ruixue, Liangzhi, Crystal Fuji, and Guifei. Specifically, butanol, hexanol, 6-methyl-5-heptene-2-ol, 2-heptanol, 2-methyl-1-butanol, and 2-ethylhexanol contributed “green, liquor, fruity” notes to the apples. Although 3-methylthiopropanol was present only at trace concentrations, it was identified as an important characteristic compound due to its high AI. Acid compounds were rarely identified as aroma-active compounds due to their high odor thresholds and low concentrations in apples; however, these acids may synergize with or mask other aroma compounds, thereby contributing to the unique flavor profile of the apples. Most acids contributed “sour and waxy” notes to the apple flavor. Sesquiterpenes were detected in low diversity and abundance, contributing “citrus, herbal, and green” notes to the apple aroma. Vanillin (AI: 3.8) mainly contributed to the “sweet” note of apples. Using SBSE, a range of furan compounds were detected in the apples, including furfuryl alcohol (AI: 3.6 to 4.5), furfural (AI: 3.4 to 3.6), and 5-methylfurfural (AI: 3.5 to 3.7). These compounds contributed “burnt sugar” notes to the characteristic aroma of the apples.

Nevertheless, there were two drawbacks in the GC-O sniffing experiment. Firstly, different evaluators provided distinct aroma descriptions and intensity scores for the same volatile compound due to the differences in individual sniffing sensitivity, which inevitably introduced human error [33]. Furthermore, the contribution of aroma compounds detected by GC-O was based on an air matrix, which differed from the actual apple solution matrix. This discrepancy can alter the odor thresholds of the compounds in question. Therefore, it was necessary to further validate the key characteristic aroma compounds using OAV calculations [3].

### 3.2. Quantitative Analysis and OAVs of Volatile Compounds

**Figure 1:**
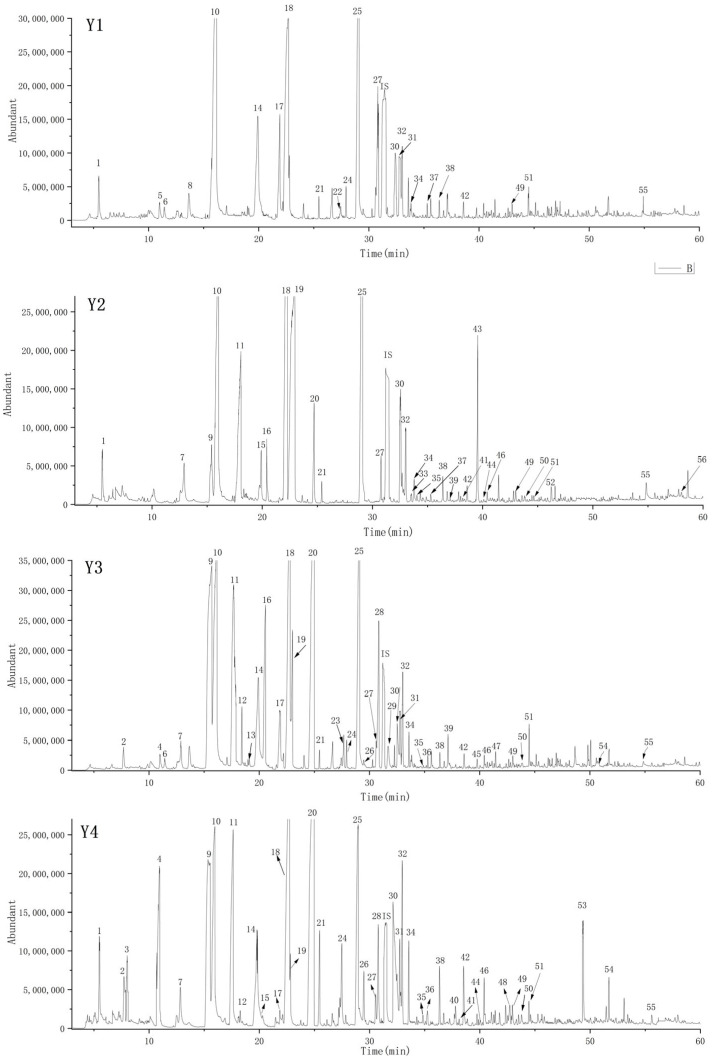
Total ion chromatogram (TIC) of the Y1 (Ruixue), Y2 (Liangzhi), Y3 (Crystal Fuji), and Y4 (Guifei) samples in the HP-INNOWAX column. The numbers (1–56) in the peaks refer to the identified aroma compounds in Table 1. IS is the peak of the internal standard 2-octanol.

As shown in Table 2 and Figure 1, a total of 56 volatile compounds were identified by SBSE combined with GC-MS in four apple samples. It could be seen that the SBSE method has the characteristics of larger adsorption capacity and larger valid extraction volume. The standard curves were established for quantitative analysis of volatile compounds, which is necessary to obtain insight into the significance of individual aroma compounds for the overall aroma of apples. The determination coefficients (R2), quantifying ions, and standard curves of all volatile compounds are summarized in Table 2. Quantitatively, butyl acetate (689–6562 μg/kg), hexyl acetate (3402–9382 μg/kg), 2-methylbutyl acetate (1456–6317 μg/kg), 1-butanol (407–7966 μg/kg), 2-methyl-1-butanol (1900–13,637 μg/kg), 1-hexanol (27,296–31,521 μg/kg), hexanal (19,686–43,381 μg/kg), and so on revealed relatively higher concentrations. Most of these compounds have been reported as the main volatile compounds in apples [27,28,29,30].

However, the concentration of individual aroma compounds was incapable of evaluating their significant contribution to the overall aroma of the apples. Thus, the concentration of each compound must be paired with the corresponding odor threshold (OT) in the corresponding matrix. The odor active values (OAVs) were utilized to better indicate how much each compound contributes to the overall aroma. Further screened by the OAV, as shown in Table 3, 14, 23, 30, and 31 volatile compounds with an OAV ≥ 1 were identified in Ruixue, Liangzhi, Crystal Fuji, and Guifei apples, respectively. Among them, hexyl acetate (OAV: 97–269), 2-methylbutyl acetate (OAV: 46–619), butyl acetate (OAV: 95–904), (E)-2-hexenal (73–293), hexanal (OAV: 29–64), 1-hexanol (OAV: 10–15), butanoic acid (OAV: 155), β-damascenone (OAV: 23), pentyl acetate (OAV: 69–112), ethyl butanoate (OAV: 29–39), 2-methyl-1-butanol (OAV: 5–35), and so on presented higher OAVs than other compounds. Thus, these compounds were regarded as critical contributors to the unique aroma of apples.

Ester compounds are an important factor influencing the flavor of apples [10], whether in terms of quantity or content, and this category accounted for the largest proportion. Specifically, butyl acetate, hexyl acetate, and 2-methylbutyl acetate were considered as characteristic esters widely distributed in many apple samples, which were formed by the condensation of acetyl-CoA, 2-methyl-1-butanol, or 1-hexanol, respectively, under the action of alcohol-acyltransferase [32,34].

In addition, aldehydes had lower content and fewer types than esters in ripe apples. Still, there were several characteristic aldehydes, especially the two C6-aldehydes (hexanal and (E)-2-hexenal), which protect the plants from infection and pest control when plant tissues are destroyed. Alcohol compounds were another important class of apple aroma compounds. In this study, butanol, hexanol, 2-methyl-1-butanol, and other alcohols with OAVs ≥ 1 contributed significantly to apple flavor. Notably, citronellol (OAV: 2) and α-terpineol (OAV: 3) were first detected in these apples. Moreover, 3-methylthiopropanol (OAV: 1–2) was recognized as a characteristic compound in apples despite its concentration being at a trace level, which contributed to the “sulfur” note. While it might have synergistic effects with other aroma compounds and make a greater contribution to the aroma profile of apples, further research will be carried out in subsequent experiments.

Acid compounds were rarely identified as aroma-active compounds in apples due to their high thresholds and low concentrations. However, they played an important role in shaping the unique flavor profile of apples. In this study, butanoic acid exhibited a notably high OAV (OAV: 155) and contributed “sour and fat” notes. Lactones have rarely been reported in apples. γ-Hexalactone (OAV: 1), γ-octalactone (OAV: 1), γ-undecanolactone (OAV: 2), and γ-decalactone (OAV: 2) had relatively high OAVs. The lactones in fruits were formed from saturated fatty acids by dehydrogenation, epoxidation, hydration hydroxylation, b-oxidation shortening, and internal esterification with hydroxyacetyl-coA [35]. Collectively, these compounds provide significant contributions to the overall aroma profile of the apples.

### 3.3. Volatile Compounds in Four Apple Samples Identified by HS-GC-IMS

With the advantages of high sensitivity, low environmental requirements, and data visualization, GC-IMS could effectively separate ions according to the mobility rate of their ions under atmospheric pressure, especially for the separation of isomers and isobaric compounds [2,22]. The volatile compounds of Ruixue, Liangzhi, Crystal Fuji, and Guifei apples were analyzed via GC-IMS for the first time, as detailed below.

#### 3.3.1. Profile Analysis by GC-IMS

**Figure 2:**
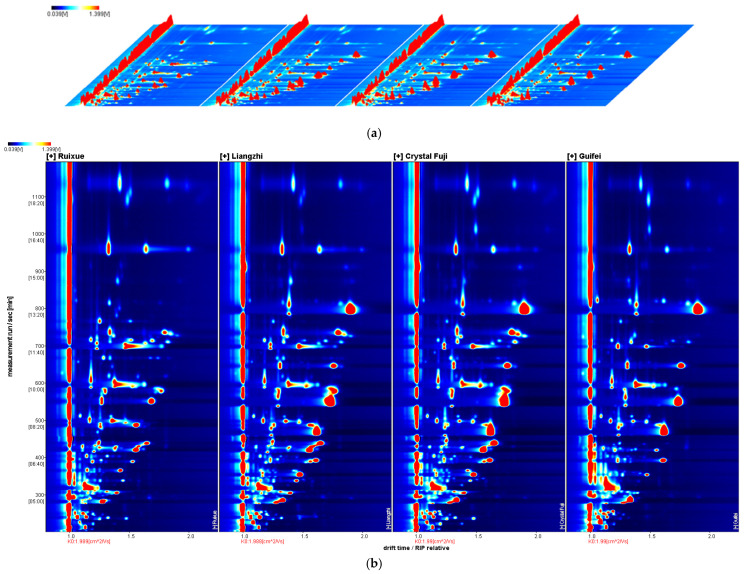

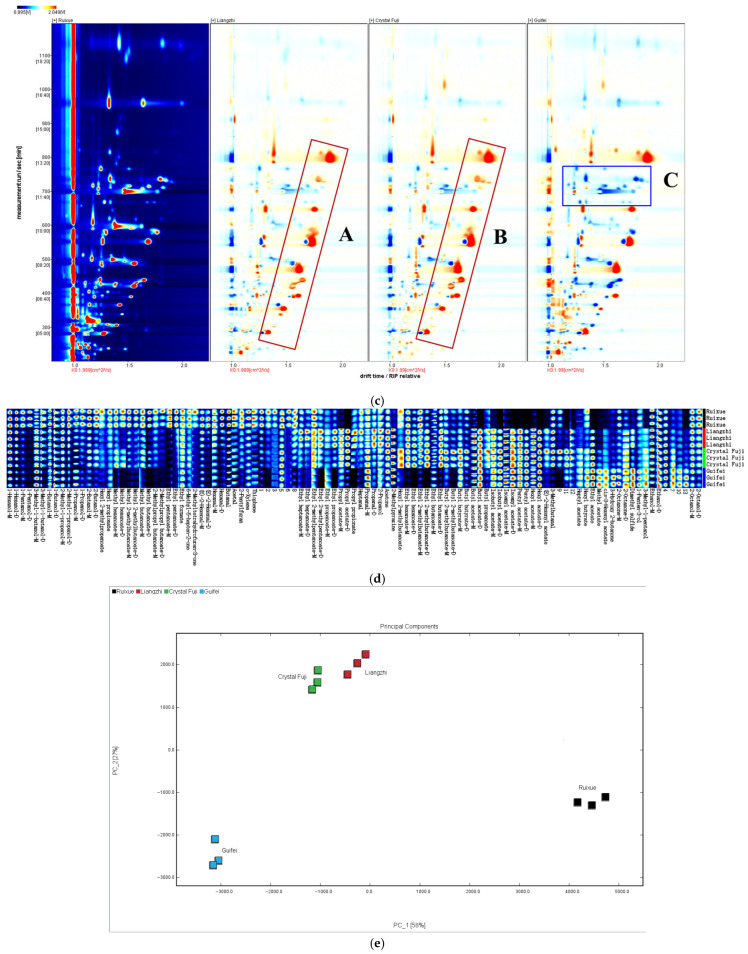

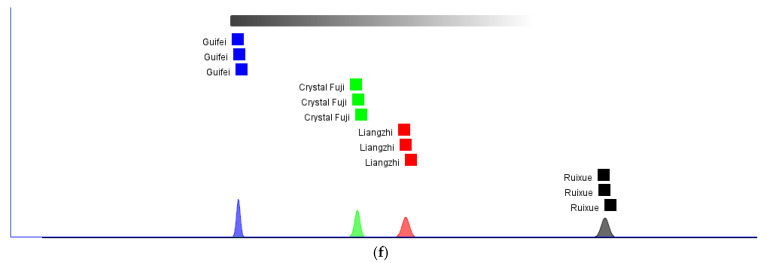
The results of Ruixue, Liangzhi, Crystal Fuji, and Guifei apples analyzed by GC−IMS. (**a**) 3D chromatogram; (**b**) 2D chromatogram; (**c**) discrepancy image; and (**d**) fingerprints. Note: The bright spot is a volatile component, and its hue spans from blue to red, indicating the concentration of a compound from lesser to greater. Each row represents all the signal peaks selected in a sample, and each column denotes the signal peaks of the same volatile compounds in different apple samples. (**e**) PCA results; (**f**) nearest neighbor analysis.

The differential three-dimensional (3D) topographic plots of the volatile compounds in apples are shown in Figure 2a. The Y-axis, X-axis, and Z-axis represent the retention time of the gas chromatography, the ion migration time, and the peak intensity of each volatile component in the sample, respectively. The corresponding two-dimensional (2D) plots (Figure 2b) were obtained to more intuitively compare the differences in volatile organic compounds in various samples. The background of the entire figure is blue, and the red vertical line at horizontal coordinate 1.0 is the reaction ion peak (RIP), which required normalization processing. The vertical coordinate represents the retention time (s) of the gas chromatography, and the horizontal coordinate represents the ion migration time (normalized treatment). Each point on either side of the RIP represents a volatile organic compound. The colors indicate the relative concentration of the individual component. As shown in Figure 2c, taking Ruixue as a reference, and removing the signal peak of Ruixue from the spectrum, the spectra showing the different components were obtained. The blue area (C) shows that the compound in Guifei apples had a lower concentration than in Ruixue apples, while the red area (A, B) indicates that the compound in Liangzhi and Crystal Fuji apples had a greater relative content than in Ruixue apples. The darker the color, the greater the difference.

#### 3.3.2. Differences in Aroma-Active Compounds in Different Samples

In order to intuitively and comprehensively distinguish the differences among volatile components in the four samples, software was used to extract all volatile compounds in the spectrum to form the fingerprint (Figure 2d). The composition of volatile compounds in the four samples was significantly different. As shown in Table 4 and Figure 2d, a total of 59 typical compounds were detected from four different apples, including 31 esters, 11 alcohols, 7 aldehydes, 5 ketones, 2 sulfur compounds, 1 furan, 1 olefin compound, and 1 pyrazine, which were identified in the NIST 2014 and IMS databases. Some aroma compounds could appear as different product ions, such as monomers and dimers, due to their concentrations in HS-GC-IMS. The drift times of these volatiles were diverse, but their retention times were similar. Moreover, these products could be observed not only through multiple signals of individual compounds that form adducts between neutral molecules and ions, but also through the drift regions. In this study, 31 volatiles were revealed in dimers, observed owing to their high concentrations, and 28 volatile compounds were identified in monomers, as shown in Figure 2d.

According to the above analysis results, the volatile components in the four different varieties of apples were basically similar, while the concentration levels were significantly different. To gain insight into the differences between the volatile compounds of Ruixue, Liangzhi, Crystal Fuji, and Guifei apples, PCA and nearest neighbor analysis were carried out for multivariate statistical analysis [2,36], and the results are shown in Figure 2e,f. As shown in Figure 2e, the variance contributions of PC-1 and PC-2 were 58% and 27%, respectively, with a cumulative variance contribution of 85%, which was much larger than the confidence value of 60%. Ruixue samples were clustered in the bottom-right area, Guifei samples were mainly located in the bottom-left, and the samples of Liangzhi and Crystal Fuji were relatively close to each other and were clustered in the upper-left area (Figure 2e). The result revealed that the volatile profiles of apple samples could be distinguished clearly by GC-IMS combined with the PCA. Similarly, the nearest neighbor analysis also presented the differences in volatility profiles among the four apple samples. The reasons for these differences might be related to the geographical environment, climatic characteristics, varieties, cultivated condition, and so on. The interaction mechanism between aroma compounds such as synergistic and masking effects would be studied in more depth in the future.

As shown in Table 4 and Figure 2d, the number of esters was the highest, followed by alcohols, aldehydes, and ketones. Esters were an important factor in the richness of the ester flavor of apples [28], whether in quantity or relative content, and this category accounted for the largest proportion. Hexyl acetate, butyl acetate, 3-methylbutyl acetate, pentyl acetate, ethyl 2-methylbutanoate, ethyl butanoate, methyl butanoate, ethyl hexanoate, propyl acetate, ethyl acetate, ethyl pentanoate, and ethyl 2-methylpentanoate were considered the important characteristic esters in apples due to their higher relative concentrations, which was slightly different from the results of GC-MS. A total of 14 esters were detected simultaneously in SBSE-GC-MS and HS-GC-IMS, and the ethyl formate, methyl acetate, methyl hexanoate, heptyl acetate, (E)-2-hexenyl acetate, ethyl propanoate, propyl propanoate hexyl propanoate, ethyl pentanoate, ethyl hexanoate, methyl 2-methylbutanoate, ethyl 2-methylbutanoate, butyl 2-methylbutanoate, hexyl 2-methylbutanoate, ethyl 2-methylpentanoate, 3-methylbutyl acetate, cis-3-hexenyl acetate, and hexyl 2-methylpropanoate were identified only by GC-IMS. A consistent observation was that the content of butyl acetate was lowest in Ruixue, followed by Crystal Fuji, Liangzhi, and Guifei. This trend was consistent with the results of SBSE-GC-MS analysis, indicating that butyl acetate can serve as a potential marker for distinguishing between these apple varieties.

Although alcohols were present in relatively low concentrations, they were crucial components of the overall apple flavor profile. As shown in Table 4, the total relative content of alcohol detected in Guifei apples was significantly higher than that in Ruixue, Liangzhi, and Crystal Fuji apples. Specifically, the contents of 1-penten-3-ol, 3-methyl-1-pentanol, ethanol, 1-propanol, and 2-propanol in Guifei apples exceeded those in the other three samples. There are two additional alcohols (1-butanol and 1-hexanol) that were also identified via SBSE-GC-MS analysis. Among all the identified alcohol compounds, ethanol, 1-butanol, 3-methyl-1-butanol, 1-hexanol, and 2-methyl-1-propanol exhibited higher relative contents than the other alcohols, contributing alcoholic and green sensory notes to the apples.

Moreover, butanal, 3-methylbutanal, hexanal, and trans-2-hexenal have been identified by SBSE-GC-MS and HS-GC-IMS, while propanal, heptanal, and acetal were detected only by HS-GC-IMS. It is worth noting that hexanal and trans-2-hexenal were important odorants of apples; they had green and woody aromas and could enhance the freshness of apples. 3-Hydroxy-2-butanone and 2-methyltetrahydrofuran-3-one were detected by HS-GC-IMS, but could not be found by SBSE-GC-MS, and they contributed to the sweet and woody notes for the aroma profile of apples. Confusingly, no acid compounds were detected in apples by HS-GC-IMS, but 2-methyl pyrazine, thiophene, and dimethyl sulfide were not identified by SBSE-GC-MS. The effects of pyrazines and sulfur-containing compounds on apple flavor were further investigated in subsequent studies. According to the above analysis results, the volatile components in the four different varieties of apples were basically similar, while the concentration levels were significantly different. Notably, HS-GC-IMS enables rapid and effective differentiation of the aroma characteristics among different apple cultivars. Furthermore, the volatile fingerprints of apple samples detected from HS-GC-IMS showed that there were great differences between the analysis results of esters, alcohols, aldehydes, ketones, and other volatile compounds and those of SBSE-GC-MS, which further verified the importance of comprehensive identification of volatile components in apples by multiple instruments.

### 3.4. Aroma Recombination Model Evaluation

**Figure 3:**
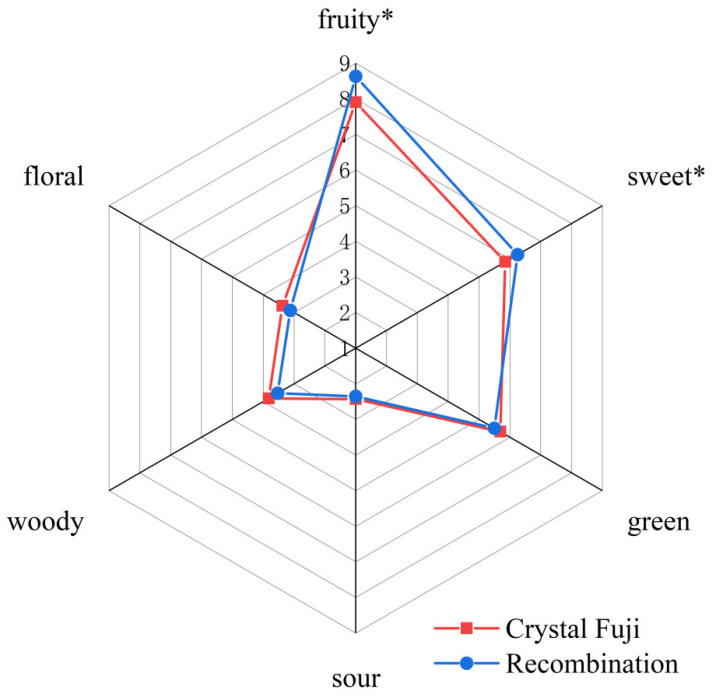
Aroma profile of Crystal Fuji apples and the aroma recombination by the sensory panel. * *p* < 0.01.

In our previous study, we conducted sensory analysis on Ruixue, Liangzhi, Crystal Fuji, and Guifei apple samples and classified the overall aroma profile of the apples into six attributes (fruity, sweet, green, floral, woody, and sour) [3]. To verify that the selected aroma-active compounds (AIs ≥ 3.5, OAVs ≥ 1) were the key substances constituting the overall aroma profile of apples, an aroma recombination experiment based on Crystal Fuji was conducted. The results (Figure 3) showed that the recombination model could effectively reproduce the characteristic aroma of apples; however, there are slight differences in the intensity of individual sensory attributes. Specifically, the recombination displayed a strengthening effect in “fruity” and “sweet” descriptors, while the “floral”, “woody”, and “green” aroma descriptors tended to weaken compared to the Crystal Fuji sample, and the “sour” descriptor was basically consistent with the original apple sample. The possible reasons for this sensory bias were due to the superposition, masking, and recombination interactions among volatiles, as well as the changed volatile release kinetics due to recombination of the matrix [37]. Moreover, the reason that the score of the “sweet” descriptor of the recombination model was weaker than that of the original sample might be that the odorant induced an enhancement of the sweet substances in the model matrix. In conclusion, the experimental results showed good similarities between the recombination model and Crystal Fuji apples, which demonstrated the successful characterization of the aroma-active compounds for the apples.

### 3.5. Correlation Analysis Between Samples, Aroma-Active Compounds, and Sensory Attributes by Using PLSR

**Figure 4:**
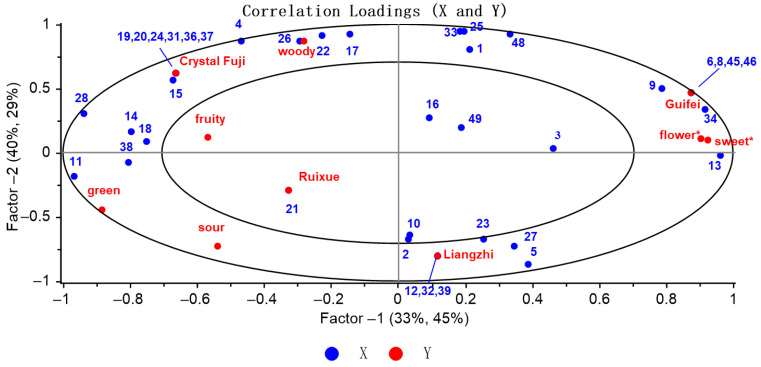
Loading plot of PLSR correlation analysis between aroma-active compounds (X-matrix) and sensory attributes of apples (Y-matrix) (thirty-nine aroma-active compounds shown in Table 3), * *p* < 0.01.

To further investigate the correlation between the scores of the sensory properties and aroma-active compounds of four apple samples, a PLSR model was established. The PLSR model presents a correlation loading graph composed of an inner ellipse and an outer ellipse, where the inner ellipse represents 50% of the explanatory variance and the outer ellipse represents 100% of the explanatory variance [38]. As shown in Figure 4, the derived PLSR model provided a two-factor model diagram, explaining 73% of the X-variable (concentration of aroma-active compounds) and 74% of the Y-variable (samples and sensory attributes), which explained the correlation between them well.

As shown in Figure 4, Ruixue, Liangzhi, Crystal Fuji, and Guifei were, respectively, distributed in four quadrants, which were mutually independent and relatively scattered. Moreover, among all sensory attributes, except for the “fruity” descriptor, the rest were distributed between the inner and outer ellipse. The six noteworthy descriptors were correlated with aroma-active compounds. From the PLSR correlation plot, Crystal Fuji was related with the “woody” descriptor, while Ruixue was correlated with the “fruity”, “green”, and “sour” descriptors, and Guifei was associated with “floral” and “sweet”, which were consistent with the results of sensory ananlysis in our previous apple study [3]. The “woody” attribute was related to butyl butanoate (No. 4), butyl propanoate (No. 17), butanal (No. 22), and 1-butanol (No. 26). The “fruity” descriptor was correlated with ethyl butanoate (No. 14), methyl butanoate (No. 18), and phenethyl alcohol (No. 38). Moreover, the “green” attribute was associated with 5-methyl furfural (No. 11). It was found that butanoic acid (No. 6), β-damascenone (No. 8), butyl acetate (No. 9), pentyl acetate (No. 13), furfuryl alcohol (No. 34), γ-decalactone (No. 45), and vanillin (No. 46) had a significant impact on the “flower” and “sweet” attributes of Guifei. Furthermore, Crystal Fuji had a strong correlation with D-limonene (No. 15), 2-methylpropyl butanoate (No. 19), hexyl butanoate (No. 20), 2-heptanol (No. 24), γ-hexalactone (No. 31), hexyl 2-methylbutanoate (No. 36), and citronellol (No. 37). Liangzhi was related with 2-methylbutyl acetate (No. 2), 1-hexanol (No. 5), trans-2-hexenal (No. 10), octyl acetate (No. 12), 2-methyl-1-butanol (No. 23), phenylacetaldehyde (No. 27), γ-octalactone (No. 32), and α-terpineol (No. 39). Ruixue was only strongly correlated with ethyl heptanoate (No. 21). The result indicated that the correlations of different aroma-active compounds vary among different samples, which could be used as a discriminator in the aroma analysis of apples to distinguish apple varieties. Combined with the correlation analysis of aroma evaluation by PLSR, it was further clarified that 39 aroma-active compounds had correlation with the six sensory attributes and had a significant impact on the overall apple flavor.

## 4. Conclusions

In summary, 59 volatiles were detected by GC-IMS technology, which, combined with PCA, confirmed that there were significant differences in the aroma compounds of four apple samples. Meanwhile, 56 volatile aroma compounds in apples were qualitatively and quantitatively analyzed by SBSE-GC-MS, and 39 aroma-active compounds were screened with high AI values and OAVs. In addition, the olfactory thresholds of the 56 aroma compounds were determined by a 3-AFC method and measured in the apple model solution. Aroma recombination experimentation displayed a strengthening effect in “fruity” and “sweet” descriptors, while the “floral”, “woody”, and “green” aroma descriptors tended to weaken compared to the Crystal Fuji sample, which confirmed that the 39 aroma-active compounds were the main components constituting the characteristic aroma profile of apples. Finally, the PLSR analysis showed that Crystal Fuji exhibited the greatest correlation with the “wood” attribute, and Ruixue was related with the “fruity”, “green”, and “sour” attributes, while butanoic acid, β-damascenone, butyl acetate, pentyl acetate, furfuryl alcohol, γ-Decalactone, and Vanillin had a significant impact on the “flower” and “sweet” attributes of Guifei. The experimental results would provide theoretical support for improving the flavor of apples and offer new ideas for guiding new apple varieties.

## Figures and Tables

**Table 1:** The aromatic compounds in apple were identified by GC-O.

NO.	Compounds	RT ^A^		RI ^B^		RI ^C^		Basis of ID ^D^	Aroma Description ^E^	AI ^F^							
		HP-INNOWAX	DB-5	HP-INNOWAX	DB-5	HP-INNOWAX	DB-5			Ruixue	RSD (%)	Liangzhi	RSD (%)	Crystal Fuji	RSD (%)	Guifei	RSD (%)
1	Acetaldehyde	5.66	4.12	707	<600	707	<600	RI, AD, Std	pungent, fresh, green	3.1	1.13	3.7	3.11			3.3	3.24
2	Butanal	7.67	4.95	864	593	860	<600	RI, AD, Std	cocoa, green, fermented					3.7	2.38	3.8	3.85
3	Ethyl acetate	7.98	5.38	869	628	869	630	RI, AD, Std	pineapple, grape							3.3	4.55
4	Propyl acetate	10.4	6.68	957	713	958	710	RI, AD, Std	fruity, tutti-frutti, and honey					4.1	2.79	4.2	7.05
5	3-Methylbutanal	10.9	5.85	967	664	965	660	RI, AD, Std	green, fatty, cocoa	2.8	1.51						
6	Methyl butanoate	11.4	7.02	975	730	958	732	RI, AD, Std	fruity, apple-like and cheese	3.6	1.47			3.5	4.41		
7	2-Methylpropyl acetate	12.6	8.04	1000	772	1002	772	RI, AD, Std	fruity with a banana note			4.1	2.53	4.4	1.3	3.9	1.35
8	Ethyl butanoate	13.64	9.03	1024	808	1022	810	RI, AD, Std	fruity, sweet	5.8	2.56	5.6	1.62				
9	Butyl acetate	15.41	9.30	1068	816	1068	819	RI, AD, Std	sweet, ripe banana	5.2	2.87	8.8	1.45	7.7	2.23	8.1	1.71
10	Hexanal	15.97	8.98	1080	807	1080	808	RI, AD, Std	green, apple with a fresh	7.2	1.81	6.2	1.61	5.5	1.06	6.1	0.94
11	2-Methylbutyl acetate	18.04	10.9	1126	861	1126	862	RI, AD, Std	sweet, banana, juicy fruit			8.8	0.67	7.3	0.845	6.5	0.879
12	Butyl propanoate	18.43	12.76	1162	908	1160	909	RI, AD, Std	sweet, banana, tropical fruit					4.2	2.7	3.9	2.5
13	2-Methylpropyl butanoate	19.1	14.99	1148	961	1148	962	RI, AD, Std	sweet, fruity, bubble gum					3.5	2.78		
14	1-Butanol	19.76	6.02	1136	676	1130	678	RI, AD, Std	banana fusel, green	5.9	5.26			6.8	3.58	5.9	1.46
15	Pentyl acetate	19.91	12.92	1168	913	1160	915	RI, AD, Std	sweet, pear, overripe banana			6.1	1.59			6.9	1.55
16	D-Limonene	20.55	17.7	1183	1018	1183	1020	RI, AD, Std	citrus, herbal, camphor			4.3	3.15	6.5	1.82		
17	Butyl butanoate	21.76	16.6	1205	993	1210	993	RI, AD, Std	sweet fruity, elderberry fatty	6.1	1.23			5.3	0.69	5.9	0.72
18	(E)-2-hexenal	22.69	10.8	1202	860	1202	861	RI, AD, Std	fresh green, leafy	6.5	1.52	7.6	1.23	6.9	0.83	7.1	1.39
19	2-Methyl-1-butanol	22.78	7.26	1195	742	1198	742	RI, AD, Std	alcoholic, fatty greasy cocoa			6.2	1.92	4.1	2.44	4.2	1.39
20	Hexyl acetate	24.89	17.6	1265	1017	1265	1018	RI, AD, Std	fruity, green, sweet			7.3	1.4	7.8	0.691	8.1	0.71
21	2-Octanone	25.58	25.5	1293	1180	1293	1180	RI, AD, Std	dairy, waxy, woody	2.5	1.63	2.7	1.55	2.2	1.79	2.8	1.51
22	Ethyl heptanoate	27.31	22.1	1317	1108	1317	1110	RI, AD, Std	fruity	4.2	2.96						
23	2-Heptanol	27.44	11. 9	1309	888	1309	888	RI, AD, Std	fresh lemon-like, herbal note					3.8	1.53		
24	6-Methyl-5-hepten-2-one	27.89	27.9	1332	1232	1322	1228	RI, AD, Std	fruity, fresh green note	3.1	4.41			3.2	2.63	3.1	1.63
25	1-Hexanol	28.95	11.6	1346	881	1346	882	RI, AD, Std	fermented, green, apple-skin	5.1	1.14	6.3	4.8	5.1	1.21	5.2	4.5
26	2-Ethylhexyl acetate	29.48	23.7	1374	1144	1374	1146	RI, AD, Std	earthy herbal					2.8	8.33	3.3	4.55
27	(E,E)-2,4-Hexadienal	30.62	13.3	1388	921	1388	923	RI, AD, Std	sweet, green, waxy, spicy	3.7	5.71	3.6	2.86	3.6	2.7	3.5	2.78
28	Hexyl butanoate	30.86	25.9	1403	1188	1403	1190	RI, AD, Std	green, soapy and fruity					4.2	2.56		
29	Hexyl 2-methylbutanoate	31.67	28.78	1421	1251	1417	1250	RI, AD, Std	green, waxy, unripe fruity					3.9	1.55		
30	Acetic acid	32.43	5.61	1446	646	1447	650	RI, AD, Std	sharp, pungent, sour	2.1	7.16	3.6	3.09	1.7	5.56	3.9	2.84
31	6-Methyl-5-hepten-2-ol	32.82	16.16	1453	985	1453	986	RI, AD, Std	sweet oily green coriander	3.5	4			2.8	2.09	3.7	3.85
32	Furfural	33.02	10.1	1460	835	1468	836	RI, AD, Std	bread, almond, sweet	3.4	1.68	3.6	1.59	3.5	3.24	3.6	3.15
33	Octyl acetate	33.38	26.4	1476	1199	1474	1199	RI, AD, Std	green, waxy					5.1	1.85		
34	2-Ethylhexanol	33.59	18.3	1482	1031	1482	1032	RI, AD, Std	sweet fatty fruity	2.1	4.76	2.2	4.55	1.9	10.1	2.3	7.16
35	Benzaldehyde	34.89	15.4	1520	968	1520	969	RI, AD, Std	oily, almond, woody			1.9	5.26	2.2	4.55	2.1	2.79
36	Linalool	35.3	21.8	1535	1103	1535	1102	RI, AD, Std	flower, lavender					4.5	1.27	4.7	2.08
37	Propanoic acid	35.32	6.38	1538	696	1540	697	RI, AD, Std	pungent sour, cheese	2.9	1.46	2.5	1.63				
38	5-Methyl furfural	36.39	15.3	1574	967	1576	967	RI, AD, Std	burnt sugar	3.6	2.7	3.6	1.59	3.5	2.78	3.7	2.63
39	Hexyl hexanoate	37.05	36.0	1583	1388	1583	1390	RI, AD, Std	fruity, green with tropical			1.9	5.01	2.6	3.85		
40	Butanoic acid	37.66	10.2	1632	843	1632	846	RI, AD, Std	sour, cheese							6.3	1.35
41	Phenylacetaldehyde	38.367	18.9	1646	1045	1640	1041	RI, AD, Std	fatty, fruity, floral			3.2	1.36			2.8	1.43
42	Furfuryl alcohol	38.59	10.9	1651	862	1651	862	RI, AD, Std	burnt, sweet	3.8	1.57	3.8	2.7	3.6	3.3	4.5	4.37
43	α-Terpineol	39.56	26.2	1691	1195	1691	1193	RI, AD, Std	mint, green			3.8	2.56				
44	3-Methylthiopropanol	40.01	16.06	1706	983	1706	982	RI, AD, Std	sulfur			3.3	2.7			3.9	2.5
45	γ-Hexalactone	40.039	16.8	1713	998	1713	995	RI, AD, Std	sweet, green					3.5	2.86		
46	Benzyl acetate	40.35	23.4	1718	1139	1718	1140	RI, AD, Std	fruity, sweet, floral			3.1	2.71	3.6	6.25	3.1	8.81
47	Citronellol	41.13	28.50	1756	1245	1758	1245	RI, AD, Std	floral					3.8	1.59		
48	β-Damascenone	42.68	36.09	1822	1390	1820	1391	RI, AD, Std	sweet, floral							5.8	2.62
49	Hexanoic acid	43.01	15.68	1833	976	1823	978	RI, AD, Std	rancid	3.2	1.46	3.1	2.44	3.2	2.33	3.5	2.53
50	Phenethyl acetate	43.81	27.79	1871	1229	1861	1230	RI, AD, Std	fruity, with jasmine floral			2.1	4.76	2.4	4.17	2.1	4.76
51	Phenethyl alcohol	44.57	22.38	1906	1116	1900	1126	RI, AD, Std	floral, sweet	3.5	1.63	3.8	1.68	3.5	2.94	3.6	1.62
52	γ-Octalactone	46.33	29.01	1938	1256	1938	1253	RI, AD, Std	sweet			3.7	2.71				
53	γ-Decalactone	49.31	39.70	2140	1466	2140	1468	RI, AD, Std	sweet, milk							3.9	3.13
54	γ-Undecalactone	51.01	43.38	2236	1558	2228	1558	RI, AD, Std	sweet, fruity					3.9	2.63	3.8	1.51
55	Benzoic acid	54.85	24.98	2433	1171	2435	1172	RI, AD, Std	sour juice	1.9	5.26	2.1	4.76	2.6	3.85	2.1	4.76
56	Vanillin	58.04	37.47	2575	1418	2579	1419	RI, AD, Std	vanilla			3.8	2.56				

^A^ The retention time of volatile compounds on HP-Innowax and DB-5 columns. ^B^ The retention index of volatile compounds on HP-Innowax and DB-5 columns. ^C^ The retention index of volatile compounds on HP-Innowax and DB-5 columns, referring to the database (https://webbook.nist.gov/chemistry/ (accessed on 6 October 2024)). ^D^ Method of identification—AD: aroma descriptor, RI: retention index, and Std: confirmed by authentic standards. ^E^ Descriptors of the actual smell. ^F^ Aroma intensity of GC-O.

**Table 2:** External standard curves and concentrations of aroma compounds in ‘Ruixue’, ‘Liangzhi’, ‘Crystal Fuji’, and ‘Guifei’ apples.

Compounds	RI ^a^		Identification ^b^	Quantitative	Standard Curves ^c^	R^2^	Concentration (μg/kg) ^d^			
	Innowax	DB-5		Ions			Ruixue	Liangzhi	Crystal Fuji	Guifei
Esters										
Ethyl acetate	869	628	RI, Std, MS	43	y = 0.068x + 0.235	0.995				869 ± 1.2
Propyl acetate	957	713	RI, Std, MS	43	y = 0.269x + 0.213	0.998			2950 ± 3.5	3730 ± 0.9
Butyl acetate	1068	816	RI, Std, MS	43	y = 0.146x + 1.95	0.999	689 ± 3.3	3035 ± 0.3	3112 ± 4.8	6562 ± 1.4
2-Methylpropyl acetate	1000	772	RI, Std, MS	43	y = 0.556x + 0.186	0.999		126 ± 4.8	140 ± 0.8	103 ± 4.9
Pentyl acetate	1168	913	RI, Std, MS	43	y = 0.341x − 0.049	0.997		203 ± 2.5		330 ± 2.1
Hexyl acetate	1265	1024	RI, Std, MS	43	y = 0.074x − 0.074	0.997		3402 ± 1.7	9382 ± 0.6	8866 ± 0.6
Octyl acetate	1474	1199	RI, Std, MS	70	y = 0.009x + 0.006	0.991		447 ± 2.3		
Benzyl acetate	1718	1139	RI, Std, MS	108	y = 1.18x + 0.062	0.998		35.21 ± 2.2	34.22 ± 2.7	28.39 ± 5.1
Phenethyl acetate	1871	1229	RI, Std, MS	104	y = 2.99x + 0.061	0.996		9.942 ± 4.7	9.972 ± 1.1	8.852 ± 2.3
2-Ethylhexyl acetate	1374	1144	RI, Std, MS	43	y = 0.159x − 0.002	0.999			376.7 ± 1.4	1190 ± 0.3
Butyl propanoate	1132	910	RI, Std, MS	71	y = 0.471x − 0.086	0.999			175 ± 1.5	105 ± 4.8
Methyl butanoate	975	730	RI, Std, MS	43	y = 0.423x + 0.093	0.995	110 ± 2.1		67.55 ± 1.1	
Ethyl butanoate	1024	808	RI, Std, MS	71	y = 0.239x + 0.142	0.996	291 ± 1.9		214 ± 2.3	
Butyl butanoate	1205	993	RI, Std, MS	71	y = 0.117x + 0.045	0.997	175 ± 0.5		340 ± 1.2	175 ± 1.3
2-Methylpropyl butanoate	1148	961	RI, Std, MS	71	y = 1.08x − 0.066	0.991			35.62 ± 1.4	
Hexyl butanoate	1403	1188	RI, Std, MS	71	y = 0.036x + 0.019	0.998			1071 ± 0.8	
Hexyl hexanoate	1583	1388	RI, Std, MS	117	y = 0.137x − 0.003	0.998		11.54 ± 2.6	56.71 ± 0.8	
Ethyl heptanoate	1317	1108	RI, Std, MS	88	y = 0.211x − 0.011	0.991	30.81 ± 1.5			
2-Methylbutyl acetate	1126	861	RI, Std, MS	43	y = 0.132x + 0.384	0.992		6317 ± 0.8	1456 ± 1.9	4700 ± 0.4
Hexyl 2-methylbutanoate	1421	1251	RI, Std, MS	57	y = 0.345x + 0.002	0.999			413.1 ± 2.7	
Alcohols										
1-Butanol	1136	676	RI, Std, MS	56	y = 0.015x + 0.076	0.991	407 ± 0.2		7966 ± 0.2	4253 ± 1.2
2-Methyl-1-butanol	1195	762	RI, Std, MS	57	y = 0.023x + 0.183	0.997		13,637 ± 1.6	1900 ± 2.1	3178 ± 1.7
1-Hexanol	1346	881	RI, Std, MS	56	y = 0.097x + 0.756	0.998	28,781 ± 1.8	31,521 ± 0.5	27,296 ± 0.8	21,699 ± 0.8
2-Ethylhexanol	1482	1031	RI, Std, MS	57	y = 0.975x + 0.231	0.998	111 ± 4.6	115 ± 4.4	106 ± 3.4	90.29 ± 0.6
2-Heptanol	1309	888	RI, Std, MS	45	y = 0.976x + 0.442	0.997			213 ± 2.4	
6-Methyl-5-hepten-2-ol	1453	985	RI, Std, MS	95	y = 0.251x + 0.493	0.996	954 ± 1.1		356 ± 1.4	928 ± 3.8
Linalool	1535	1103	RI, Std, MS	71	y = 0.633x + 0.012	0.997			717 ± 2.1	733 ± 1.4
Citronellol	1756	1245	RI, Std, MS	69	y = 0.263x + 0.001	0.999			29.3 ± 5.3	
Phenethyl alcohol	1906	1116	RI, Std, MS	91	y = 0.345x + 0.044	0.998	54.03 ± 3.4	55.44 ± 5.9	56.51 ± 1.8	52.57 ± 3.1
α-Terpineol	1691	1195	RI, Std, MS	43	Y = 0.165x + 0.038	0.992		9239 ± 2.7		
3-Methylthiopropanol	1706	983	RI, Std, MS	106	y = 2.278x − 0.001	0.995		4.8 ± 5.6		6.21 ± 8.1
Aldehydes										
Acetaldehyde	707	<600	RI, Std, MS	44	y = 0.015x + 0.015	0.996	837 ± 2.2	859 ± 2.9		7159 ± 2.1
Butanal	864	593	RI, Std, MS	44	y = 0.082x + 0.022	0.991			98.91 ± 1.1	43.63 ± 6.1
3-Methylbutanal	967	664	RI, Std, MS	58	y = 0.252x + 0.016	0.997	2.13 ± 4.6			
Hexanal	1080	807	RI, Std, MS	44	y = 0.078x + 0.876	0.994	43,381 ± 1.1	22,732 ± 2.9	19,686 ± 2.6	41,227 ± 3.6
Benzaldehyde	1520	967	RI, Std, MS	106	y = 1.16x + 0.039	0.998		15.66 ± 6.4	16.29 ± 5.6	14.96 ± 5.1
Phenylacetaldehyde	1646	1045	RI, Std, MS	91	y = 0.429x + 0.001	0.996		54.93 ± 3.8		13.21 ± 7.7
Trans-2-hexenal	1202	860	RI, Std, MS	41	y = 0.321x + 0.004	0.999	40,512 ± 3.1	161,882 ± 3.3	73,161 ± 2.8	53,240 ± 1.9
(E,E)-2,4-Hexadienal	1388	1033	RI, Std, MS	81	y = 0.475x + 0.252	0.995	228 ± 11	177 ± 4.6	252 ± 8.1	164 ± 3.1
Ketones										
2-Octanone	1293	1180	RI, Std, MS	43	y = 0.553x + 0.052	0.995	25.94 ± 9.8	12.25 ± 4.6	26.53 ± 5.7	35.51 ± 2.9
6-Methyl-5-hepten-2-one	1332	1232	RI, Std, MS	43	y = 0.708x + 0.078	0.998	51.27 ± 3.5		37.09 ± 4.2	45.28 ± 3.3
β-Damascenone	1822	1390	RI, Std, MS	69	y = 0.158x + 0.017	0.996				20.08 ± 3.6
Acids										
Acetic acid	1446	602	RI, Std, MS	43	y = 0.005x + 0.014	0.996	1991 ± 1.3	19,186 ± 2.9	7772 ± 1.2	36,392 ± 2.9
Propanoic acid	1538	696	RI, Std, MS	74	y = 0.077x + 0.005	0.995	22.22 ± 11	4.132 ± 2.8		
Butanoic acid	1632	843	RI, Std, MS	60	y = 0.031x + 0.003	0.996				113 ± 4.5
Hexanoic acid	1833	1291	RI, Std, MS	60	y = 0.108x + 0.007	0.991	5.960 ± 15	9.082 ± 5.9	12.52 ± 4.9	27.22 ± 2.5
Benzoic acid	2433	1309	RI, Std, MS	105	y = 2.12x − 0.015	0.996	4.76 ± 8.9	9.891 ± 1.5	5.381 ± 1.2	4.821 ± 7.8
Lactones										
γ-Hexalactone	1713	998	RI, Std, MS	85	y = 0.086x + 0.013	0.998			54.45 ± 0.9	
γ-Octalactone	1938	1256	RI, Std, MS	85	y = 1.87x + 0.041	0.992		9.531 ± 6.4		
γ-Decalactone	2140	1466	RI, Std, MS	85	y = 3.13x + 0.017	0.998				191 ± 3.5
γ-Undecalactone	2236	1558	RI, Std, MS	85	y = 3.11x − 0.036	0.997			63.12 ± 1.7	63.51 ± 4.1
Others										
D-Limonene	1183	1018	RI, Std, MS	68	y = 0.005x − 0.003	0.993		1301 ± 2.2	15,612 ± 3.2	
Furfuryl alcohol	1651	862	RI, Std, MS	98	y = 0.015x + 0.004	0.991	150 ± 1.5	128 ± 2.8	59.33 ± 1.7	559 ± 7.2
Furfural	1460	835	RI, Std, MS	96	y = 0.008x + 0.052	0.993	2095 ± 0.5	3412 ± 1.6	3236 ± 1.6	4879 ± 5.4
5-Methyl furfural	1574	967	RI, Std, MS	110	y = 0.428x + 0.136	0.997	140 ± 5.5	138 ± 4.5	147 ± 3.5	117 ± 4.6
Vanillin	2575	1418	RI, Std, MS	151	y = 0.231x + 0.006	0.998		101.2 ± 5.3		

^a^ The retention index of volatile compounds on DB-5 and HP-Innowax columns. ^b^ Method of identification: RI: retention index, Std: confirmed by authentic standards, and MS: mass spectrometry. ^c^ Calibration curves were constructed by the following formula: Ax/Ai = a(Cx/Ci) + b. A denotes the peak area and C denotes the concentration, and x and i denote the standard compound and inter standard, respectively. ^d^ Concentrations (μg/L) of volatile compounds quantified by SBSE.

**Table 3:** Odor thresholds and OAVs of aroma-active compounds in four apples.

	Compounds	Aroma Descriptors ^A^	Threshold ^B^	OAV			
			(μg/kg)	Ruixue	Liangzhi	Crystal Fuji	Guifei
1	Hexyl acetate	fruity, green, sweet	34.9		97	269	254
2	2-Methylbutyl acetate	sweet, fruit	10.2		619	143	460
3	1-Hexanal	green	678	64	34	29	61
4	Butyl butanoate	sweet fruity	27.5	6		12	6
5	1-Hexanol	green, fruity	2089	14	15	13	10
6	Butanoic acid	sour	0.732				155
7	Acetaldehyde	fruity, green	252	3	3		28
8	β-Damascenone	sweet, floral	0.863				23
9	Butyl acetate	sweet, fruity	7.26	95	418	429	904
10	Trans-2-hexenal	green	553	73	293	132	96
11	5-Methyl furfural	burnt sugar	65.9	2	2	2	2
12	Octyl acetate	green, waxy	40.7		11		
13	Pentyl acetate	sweet, fruity	2.94		69		112
14	Ethyl butanoate	fruity, sweet	7.51	39		29	
15	D-Limonene	fruity	177		7	88	
16	2-Methylpropyl acetate	sweet, fruity	74.5		2	2	1
17	Butyl propanoate	sweet, floral	67.3			3	2
18	Methyl butanoate	fruity	96.1	1		1	
19	2-Methylpropyl butanoate	sweet, fruity	48.6			1	
20	Hexyl butanoate	green, fruity	258.5			4	
21	Ethyl heptanoate	fruity	10.1	3			
22	1-Butanol	green	225.5	2		35	19
23	2-Methyl-1-butanol	fatty	389		35	5	8
24	2-Heptanol	green, fruity	258.8			1	
25	Linalool	flower	97.7			7	8
26	Butanal	green	37.9			3	1
27	Phenylacetaldehyde	fatty, fruity, floral	6.32		9		2
28	(E,E)-2,4-Hexadienal	sweet, green	101.2	2	2	2	2
29	Propanoic acid	sour	77.03	<1	<1		
30	Acetic acid	sour	17,093	<1	1	<1	2
31	γ-Hexalactone	sweet, green	55.2			1	
32	γ-Octalactone	sweet	8.97		1		
33	γ-Undecalactone	sweet, fruity	26.7			2	2
34	Furfuryl alcohol	burnt, sweet	60.81	2	2	1	10
35	Furfural	almond, sweet	4630	<1	1	1	1
36	Hexyl 2-methylbutanoate	green, waxy	77.8			5	
37	Citronellol	floral	12.7			2	
38	Phenethyl alcohol	floral	52.2	1	1	1	1
39	α-Terpineol	mint, green	3082		3		
40	3-Methylthiopropanol	sulfur	11.8		<1		1
41	3-Methylbutanal	green, fatty, fruity	53.1	<1			
42	2-Octanone	waxy, woody	104	<1	<1	<1	<1
43	6-Methyl-5-hepten-2-one	fruity, green	197	<1		<1	<1
44	Hexanoic acid	sour	237	<1	<1	<1	<1
45	γ-Decalactone	sweet, milk	82.7				2
46	Vanillin	sweet	78.7		1		
47	Ethyl acetate	fruity	2816				<1
48	Propyl acetate	fruity	1208			2	3
49	Benzyl acetate	green	52.1		1	1	1
50	Phenethyl acetate	fruity, floral	460		<1	<1	<1
51	2-Ethylhexyl acetate	green	918			<1	1
52	Hexyl hexanoate	sweet, fruity, green	1138		<1	<1	
53	2-Ethylhexanol	sweet, fatty, fruity	13,153	<1	<1	<1	<1
54	6-Methyl-5-hepten-2-ol	sweet, green	1403	1		<1	1
55	Benzaldehyde	sweet, woody	957		<1	<1	<1
56	Benzoic acid	sour	892	<1	<1	<1	<1

^A^ The main aroma attributes of the actual smell. ^B^ Thresholds were newly determined in the apple matrix solution.

**Table 4:** Volatile compounds in Ruixue, Liangzhi, Crystal Fuji, and Guifei apples were detected by GC-IMS.

Compounds	RT ^1^	RI ^2^	DT ^3^	Comment ^4^	Relative Content ^5^ (μg/kg, Mean ± SD, *n* = 3)			
					Ruixue	Liangzhi	Crystal Fuji	Guifei
Dimethyl sulfide	3.77	766	1.09		0.235 ± 0.003 ^d^	1.02 ± 0.07 ^b^	0.519 ± 0.06 ^c^	1.93 ± 0.2 ^a^
Propanal-M	4	797	1.071	Monomer	0.576 ± 0.008 ^bc^	0.494 ± 0.008 ^c^	0.630 ± 0.04 ^bc^	0.789 ± 0.1 ^a^
Propanal-D	4	797	1.145	Dimer	0.988 ± 0.04 ^a^	1.76 ± 0.6 ^a^	1.08 ± 0.1 ^a^	1.25 ± 0.3 ^a^
Acetone	4.17	820	1.116		0.398 ± 0.009 ^b^	0.511 ± 0.008 ^a^	0.477 ± 0.03 ^a^	0.467 ± 0.03 ^a^
Ethyl formate	4.18	823	1.049		0.190 ± 0.007 ^a^	0.157 ± 0.003 ^b^	0.168 ± 0.01 ^ab^	0.178 ± 0.02 ^ab^
Methyl acetate	4.28	834	1.195		0.018 ± 0.001 ^c^	0.0398 ± 0.002 ^c^	0.112 ± 0.007 ^b^	0.339 ± 0.05 ^a^
Butanal	4.68	882	1.28		1.79 ± 0.05 ^a^	1.274 ± 0.03 ^b^	1.26 ± 0.03 ^b^	1.41 ± 0.2 ^b^
Ethyl acetate	4.73	888	1.336		0.109 ± 0.003 ^d^	1.70 ± 0.2 ^c^	2.40 ± 0.2 ^b^	3.66 ± 0.4 ^a^
Acetal	4.88	903	1.034		0.826 ± 0.02 ^a^	0.326 ± 0.009 ^c^	0.429 ± 0.02 ^b^	0.505 ± 0.06 ^b^
3-Methylbutanal	5.08	923	1.398		0.581 ± 0.03 ^c^	0.670 ± 0.02 ^b^	0.761 ± 0.02 ^a^	0.174 ± 0.03 ^d^
2-Propanol	5.18	929	1.217		0.130 ± 0.001 ^b^	0.147 ± 0.002 ^b^	0.136 ± 0.003 ^b^	0.194 ± 0.02 ^a^
Ethanol-M	5.28	937	1.046	Monomer	0.554 ± 0.01 ^a^	0.533 ± 0.01 ^a^	0.562 ± 0.02 ^a^	0.586 ± 0.05 ^a^
Ethanol-D	5.3	938	1.127	Dimer	6.63 ± 0.07 ^b^	8.42 ± 0.4 ^b^	9.70 ± 0.4 ^b^	17.0 ± 3 ^a^
Ethyl propanoate-M	5.67	962	1.154	Monomer	0.049 ± 0.002 ^c^	0.0687 ± 0.003 ^b^	0.058 ± 0.004 ^bc^	0.0853 ± 0.01 ^a^
Ethyl propanoate-D	5.67	962	1.45	Dimer	0.282 ± 0.01 ^c^	0.966 ± 0.07 ^a^	0.499 ± 0.1 ^b^	0.029 ± 0.004 ^d^
Propyl acetate-M	5.92	978	1.168	Monomer	0.055 ± 0.003 ^c^	0.319 ± 0.007 ^a^	0.313 ± 0.010 ^a^	0.225 ± 0.02 ^b^
Propyl acetate-D	5.92	978	1.477	Dimer	0.049 ± 0.004 ^c^	4.96 ± 0.3 ^a^	4.70 ± 0.07 ^a^	1.77 ± 0.3 ^b^
Methyl butanoate-M	6.1	990	1.152	Monomer	0.286 ± 0.01 ^a^	0.163 ± 0.01 ^c^	0.215 ± 0.003 ^b^	0.162 ± 0.02 ^c^
Methyl butanoate-D	6.1	990	1.434	Dimer	1.42 ± 0.08 ^a^	0.494 ± 0.04 ^b^	1.11 ± 0.04 ^c^	0.149 ± 0.03 ^d^
Methyl 2-methylbutanoate-M	6.48	1010	1.196	Monomer	0.303 ± 0.02 ^a^	0.178 ± 0.01 ^b^	0.157 ± 0.008 ^b^	0.099 ± 0.009 ^c^
Methyl 2-methylbutanoate-D	6.48	1011	1.538	Dimer	0.608 ± 0.07 ^a^	0.401 ± 0.02 ^b^	0.450 ± 0.04 ^b^	0.336 ± 0.06 ^b^
2-Methylpropyl acetate-M	6.55	1014	1.235	Monomer	0.049 ± 0.001 ^c^	0.367 ± 0.002 ^b^	0.390 ± 0.01 ^b^	0.511 ± 0.05 ^a^
2-Methylpropyl acetate-D	6.55	1014	1.615	Dimer	0.018 ± 0.008 ^d^	2.64 ± 0.1 ^c^	3.70 ± 0.04 ^b^	5.27 ± 0.7 ^a^
Thiophene	6.6	1016	1.032		0.208 ± 0.003 ^a^	0.0990 ± 0.006 ^c^	0.0861 ± 0.01 ^c^	0.139 ± 0.02 ^b^
2-Butanol-M	6.73	1024	1.151	Monomer	0.268 ± 0.005 ^a^	0.170 ± 0.006 ^c^	0.220 ± 0.006 ^b^	0.183 ± 0.02 ^c^
2-Butanol-D	6.73	1024	1.324	Dimer	0.223 ± 0.003 ^a^	0.121 ± 0.009 ^c^	0.208 ± 0.002 ^a^	0.156 ± 0.03 ^b^
Ethyl butanoate-M	7.05	1040	1.21	Monomer	0.462 ± 0.02 ^a^	0.382 ± 0.006 ^a^	0.443 ± 0.01 ^a^	0.458 ± 0.06 ^a^
Ethyl butanoate-D	7.05	1040	1.558	Dimer	3.55 ± 0.1 ^b^	6.97 ± 0.5 ^a^	6.70 ± 3 ^a^	1.46 ± 0.3 ^b^
1-Propanol-M	7.07	1041	1.115	Monomer	0.321 ± 0.006 ^a^	0.179 ± 0.004 ^b^	0.163 ± 0.02 ^b^	0.339 ± 0.04 ^a^
1-Propanol-D	7.07	1041	1.247	Dimer	0.565 ± 0.03 ^a^	0.370 ± 0.02 ^b^	0.281 ± 0.01 ^b^	0.595 ± 0.1 ^a^
Propyl propanoate	7.2	1048	1.588		0.312 ± 0.04 ^b^	0.569 ± 0.08 ^a^	0.282 ± 0.001 ^b^	0.008 ± 0.001 ^c^
Ethyl 2-methylbutanoate-M	7.33	1054	1.25	Monomer	0.547 ± 0.01 ^a^	0.510 ± 0.01 ^a^	0.526 ± 0.03 ^a^	0.151 ± 0.02 ^a^
Ethyl 2-methylbutanoate-D	7.33	1054	1.653	Dimer	1.63 ± 0.06 ^c^	4.53 ± 0.5 ^b^	6.97 ± 0.7 ^a^	0.120 ± 0.03 ^d^
Butyl acetate-M	7.83	1076	1.239	Monomer	0.140 ± 0.005 ^c^	0.528 ± 0.02 ^ab^	0.432 ± 0.0 1^b^	0.603 ± 0.09 ^a^
Butyl acetate-D	7.85	1079	1.617	Dimer	0.138 ± 0.01 ^c^	11.8 ± 0.7 ^b^	10.7 ± 7 ^b^	23.3 ± 4 ^a^
Hexanal-M	8.12	1090	1.265	Monomer	0.362 ± 0.01 ^a^	0.334 ± 0.006 ^a^	0.247 ± 0.01 ^b^	0.300 ± 0.05 ^ab^
Hexanal-D	8.12	1090	1.56	Dimer	3.16 ± 0.04 ^a^	2.23 ± 0.2 ^b^	1.64 ± 0.01 ^b^	2.24 ± 0.5 ^b^
2-Methyl-1-propanol-M	8.37	1099	1.172	Monomer	0.561 ± 0.01 ^a^	0.561 ± 0.01 ^a^	0.478 ± 0.01 ^a^	0.547 ± 0.07 ^a^
2-Methyl-1-propanol-D	8.37	1099	1.368	Dimer	2.28 ± 0.03 ^a^	1.74 ± 0.1 ^b^	1.22 ± 0.003 ^c^	1.66 ± 0.4 ^b^
3-Methylbutyl acetate-M	9.15	1126	1.3	Monomer	0.129 ± 0.004 ^c^	0.601 ± 0.02 ^b^	0.531 ± 0.02 ^b^	0.885 ± 0.1 ^a^
3-Methylbutyl acetate-D	9.15	1125	1.747	Dimer	0.145 ± 0.015 ^c^	12.2 ± 0.7 ^b^	16.4 ± 0.7 ^a^	20.0 ± 3 ^a^
Ethyl pentanoate-M	9.18	1127	1.273	Monomer	0.792 ± 0.03 ^a^	0.108 ± 0.005 ^bc^	0.0751 ± 0.003 ^c^	0.139 ± 0.02 ^b^
Ethyl pentanoate-D	9.18	1127	1.682	Dimer	3.545 ± 0.3 ^a^	3.17 ± 0.2 ^ab^	2.29 ± 0.05 ^c^	2.70 ± 0.5 ^bc^
Ethyl 2-methylpentanoate-M	9.67	1142	1.303	Monomer	0.552 ± 0.03 ^a^	0.554 ± 0.009 ^a^	0.547 ± 0.013 ^a^	0.402 ± 0.04 ^b^
Ethyl 2-methylpentanoate-D	9.67	1142	1.766	Dimer	1.31 ± 0.205 ^c^	3.094 ± 0.203 ^a^	2.24 ± 0.07 ^b^	0.160 ± 0.05 ^d^
Butyl propanoate	9.8	1146	1.724		0.237 ± 0.03 ^b^	0.449 ± 0.03 ^a^	0.523 ± 0.07 ^a^	0.2023 ± 0.03 ^b^
1-Butanol-M	9.95	1150	1.182	Monomer	1.12 ± 0.01 ^b^	1.08 ± 0.03 ^b^	1.09 ± 0.02 ^b^	1.46 ± 0.2 ^a^
1-Butanol-D	9.95	1150	1.378	Dimer	5.80 ± 0.07 ^b^	5.61 ± 0.4 ^b^	6.54 ± 0.3 ^b^	11.5 ± 2 ^a^
2-Methylpropyl butanoate-M	10.3	1161	1.335	Monomer	0.154 ± 0.02 ^a^	0.0976 ± 0.001 ^b^	0.0833 ± 0.01 ^b^	0.029 ± 0.002 ^c^
2-Methylpropyl butanoate-D	10.3	1161	1.807	Dimer	0.124 ± 0.03 ^a^	0.0561 ± 0.002 ^b^	0.0550 ± 0.007 ^b^	0.044 ± 0.006 ^b^
1-Penten-3-ol	10.4	1165	0.947		0.096 ± 0.001 ^b^	0.0804 ± 0.002 ^b^	0.0837 ± 0.004 ^b^	0.155 ± 0.03 ^a^
Pentyl acetate-M	10.8	1175	1.312	Monomer	0.020 ± 0.004 ^c^	0.786 ± 0.02 ^b^	0.846 ± 0.01 ^b^	1.04 ± 0.1 ^a^
Pentyl acetate-D	10.8	1175	1.765	Dimer	0.0344 ± 0.01 ^d^	3.51 ± 0.2 ^c^	5.54 ± 0.1 ^b^	8.38 ± 1 ^a^
o-Xylene	10.95	1179	1.078		0.062 ± 0.003 ^a^	0.0400 ± 0.002 ^c^	0.0335 ± 0.002 ^c^	0.050 ± 0.007 ^b^
Methyl hexanoate-M	11.13	1186	1.289	Monomer	0.247 ± 0.02 ^a^	0.137 ± 0.006 ^a^	0.259 ± 0.01 ^b^	0.067 ± 0.004 ^c^
Methyl hexanoate-D	11.13	1186	1.683	Dimer	0.119 ± 0.01 ^a^	0.0360 ± 0.005 ^b^	0.120 ± 0.01 ^a^	0.022 ± 0.003 ^b^
Heptanal	11.14	1187	1.342		0.147 ± 0.01 ^a^	0.152 ± 0.018 ^a^	0.0940 ± 0.01 ^b^	0.050 ± 0.005 ^c^
3-Methyl-1-butanol-M	11.67	1211	1.232	Monomer	0.641 ± 0.01 ^b^	0.801 ± 0.02 ^a^	0.813 ± 0.02 ^a^	0.898 ± 0.1 ^a^
3-Methyl-1-butanol-D	11.67	1211	1.477	Dimer	5.68 ± 0.08 ^a^	4.95 ± 0.2 ^a^	5.06 ± 0.07 ^a^	3.37 ± 0.7 ^b^
Butyl butanoate-M	11.87	1220	1.824	Dimer	0.207 ± 0.03 ^c^	0.423 ± 0.03 ^c^	0.744 ± 0.0036 ^b^	1.023 ± 0.2 ^a^
Butyl butanoate-D	11.87	1220	1.345	Monomer	0.311 ± 0.06 ^b^	0.201 ± 0.02 ^b^	0.464 ± 0.07 ^a^	0.201 ± 0.02 ^b^
Trans-2-hexenal-M	11.95	1224	1.183	Monomer	0.305 ± 0.02 ^a^	0.258 ± 0.02 ^b^	0.171 ± 0.01 ^b^	0.229 ± 0.03 ^c^
Trans-2-hexenal-D	11.95	1224	1.521	Dimer	0.744 ± 0.1 ^a^	0.348 ± 0.06 ^b^	0.183 ± 0.006 ^c^	0.216 ± 0.05 ^bc^
2-Pentylfuran	12.05	1229	1.231		0.168 ± 0.003 ^a^	0.0850 ± 0.001 ^b^	0.0724 ± 0.002 ^c^	0.058 ± 0.007 ^d^
Butyl 2-methylbutanoate-M	12.13	1233	1.371	Monomer	0.182 ± 0.02 ^b^	0.210 ± 0.01 ^b^	0.267 ± 0.02 ^a^	0.173 ± 0.03 ^b^
Butyl 2-methylbutanoate-D	12.13	1233	1.897	Dimer	0.252 ± 0.05 ^b^	0.352 ± 0.03 ^b^	0.762 ± 0.06 ^a^	0.106 ± 0.03 ^c^
Ethyl hexanoate-M	12.27	1239	1.34	Monomer	0.8300 ± 0.03 ^b^	1.02 ± 0.03 ^a^	1.02 ± 0.03 ^a^	0.124 ± 0.01 ^c^
Ethyl hexanoate-D	12.27	1239	1.805	Dimer	1.59 ± 0.03 ^a^	3.84 ± 0.4 ^a^	4.55 ± 0.6 ^b^	0.134 ± 0.02 ^c^
1-Pentanol-M	12.75	1260	1.257	Monomer	0.457 ± 0.007 ^a^	0.1662 ± 0.01 ^bc^	0.151 ± 0.01 ^c^	0.197 ± 0.02 ^b^
1-Pentanol-D	12.75	1260	1.51	Dimer	0.31 ± 0.0003 ^a^	0.0715 ± 0.001 ^c^	0.0704 ± 0.007 ^c^	0.137 ± 0.03 ^b^
2-Methylpyrazine	12.98	1269	1.399		0.0862 ± 0.01 ^c^	0.254 ± 0.01 ^b^	0.270 ± 0.01 ^ab^	0.307 ± 0.04 ^c^
Hexyl acetate-M	13.3	1283	1.389	Monomer	0.135 ± 0.02 ^c^	1.11 ± 0.03 ^b^	1.06 ± 0.02 ^b^	1.32 ± 0.2 ^a^
Hexyl acetate-D	13.3	1283	1.897	Dimer	0.153 ± 0.04 ^c^	16.9 ± 0.8 ^b^	20.8 ± 0.5 ^b^	30.6 ± 6 ^a^
2-Methyltetrahydrofuran-3-one	13.33	1284	1.421		0.0485 ± 0.01 ^a^	0.010 ± 0.0003 ^b^	0.009 ± 0.0005 ^b^	0.014 ± 0.004 ^b^
2-Octanone-M	13.57	1294	1.335	Monomer	0.248 ± 0.01 ^b^	0.186 ± 0.01 ^c^	0.171 ± 0.008 ^c^	0.329 ± 0.05 ^a^
2-Octanone-D	13.57	1294	1.76	Dimer	0.0742 ± 0.01 ^c^	0.222 ± 0.02 ^b^	0.265 ± 0.01 ^b^	0.480 ± 0.07 ^a^
3-Hydroxy 2-butanone	13.68	1298	1.059		0.0883 ± 0.01 ^b^	0.0907 ± 0.01 ^b^	0.101 ± 0.01 ^b^	0.393 ± 0.08 ^a^
3-Methyl-1-pentanol	14.33	1319	1.329		0.007 ± 0.002 ^c^	0.0304 ± 0.003 ^b^	0.0382 ± 0.003 ^b^	0.0662 ± 0.01 ^a^
Ethyl heptanoate-M	14.57	1327	1.407	Monomer	0.296 ± 0.03 ^b^	0.395 ± 0.03 ^a^	0.268 ± 0.02 ^b^	0.164 ± 0.02 ^c^
Ethyl heptanoate-D	14.57	1327	1.914	Dimer	0.0798 ± 0.02 ^b^	0.155 ± 0.03 ^a^	0.0876 ± 0.01 ^b^	0.112 ± 0.03 ^ab^
Cis-3-hexenyl acetate	14.6	1328	1.327		0.009 ± 0.002 ^c^	0.0336 ± 0.001 ^b^	0.0424 ± 0.001 ^b^	0.112 ± 0.01 ^a^
Hexyl 2-methylpropanoate	14.75	1333	1.445		0.0464 ± 0.01 ^a^	0.0227 ± 0.001 ^b^	0.0279 ± 0.003 ^b^	0.018 ± 0.002 ^b^
(E)-2-Hexenyl acetate	15.23	1348	1.859		0.011 ± 0.004 ^b^	0.0961 ± 0.02 ^a^	0.128 ± 0.02 ^a^	0.045 ± 0.002 ^b^
Hexyl propanoate	15.23	1348	1.436		0.0768 ± 0.01 ^a^	0.06 ± 0.003 ^ab^	0.0561 ± 0.005 ^b^	0.0759 ± 0.01 ^a^
6-Methyl-5-hepten-2-one	15.25	1348	1.181		0.043 ± 0.003 ^a^	0.0304 ± 0.003 ^b^	0.0455 ± 0.002 ^a^	0.0424 ± 0.01 ^a^
1-Hexanol-M	15.97	1370	1.331	Monomer	1.86 ± 0.04 ^a^	1.73 ± 0.04 ^a^	1.71 ± 0.03 ^a^	1.66 ± 0.2 ^a^
1-Hexanol-D	15.97	1370	1.638	Dimer	2.47 ± 0.02 ^a^	2.52 ± 0.2 ^a^	2.32 ± 0.03 ^a^	2.29 ± 0.5 ^a^
Heptyl acetate	16.47	1384	1.459		0.013 ± 0.004 ^c^	0.0174 ± 0.003 ^c^	0.0441 ± 0.002 ^b^	0.054 ± 0.006 ^a^
Hexyl butanoate	18.15	1430	1.487		0.274 ± 0.04 ^b^	0.165 ± 0.02 ^c^	0.252 ± 0.02 ^bc^	0.444 ± 0.08 ^a^
Hexyl 2-methylbutanoate	18.55	1439	1.516		0.109 ± 0.02 ^a^	0.0637 ± 0.001 ^b^	0.122 ± 0.01 ^a^	0.0549 ± 0.01 ^b^

^1^ RT means the retention time (min) in the capillary GC column. ^2^ RI represents the retention index of volatile compounds calculated by using n-ketones C4–C9 (Sinopharm Chemical Reagent Co., Ltd.) as external standards. ^3^ DT represents the drift time (RIPrel) in the tube. ^4^ Denotes that the volatile component was a monomer or dimer. ^5^ The relative content of volatile compounds in apple samples was calculated based on chromatographic peak volumes and internal standard concentration. Each value was expressed as mean ± standard deviation (*n* = 3). The different letters, a–c, etc., in the same column indicate significant difference at the 0.05 level.

## Data Availability

The original contributions presented in this study are included in the article. Further inquiries can be directed to the corresponding author.

## References

[1] Qian Y., Zhang D., An Y., Zhou Q., Qian M.C. (2021). Characterization of Aroma-Active Compounds in Northern Highbush Blueberries “Bluecrop” (*Vaccinium corymbosum* “Bluecrop”) and “Elliott” (*Vaccinium corymbosum* “Elliott”) by Gas Chromatography-Olfactometry Dilution Analysis and Odor Activity Value. J. Agric. Food Chem..

[2] Feng T., Sun J., Wang K., Song S., Chen D., Zhuang H., Lu J., Li D., Meng X., Shi M. (2022). Variation in Volatile Compounds of Raw Pu-Erh Tea upon Steeping Process by Gas Chromatography-Ion Mobility Spectrometry and Characterization of the Aroma-Active Compounds in Tea Infusion Using Gas Chromatography-Olfactometry-Mass Spectrometry. J. Agric. Food Chem..

[3] Ma N., Zhu J., Wang H., Qian M.C., Xiao Z. (2024). Comparative Investigation of Aroma-Active Volatiles in (“Ruixue”, “Liangzhi”, “Crystal Fuji,” and “Guifei”) Apples by Application of Gas Chromatography-Mass Spectrometry-Olfactometry (GC-MS-O) and Two-Dimensional Gas Chromatography-Quadrupole Mass Spectrometry (GC × GC-qMS) Coupled with Sensory Molecular Science. J. Agric. Food Chem..

[4] Li J., Wang Y., Zeng C., Ma T., Fang Y., Sun X. (2025). Analysis of the Flavor Code of Newly Cultivated Excellent Apple Varieties in China Using the Sensomics. J. Agric. Food Chem..

[5] Yang S., Meng Z., Fan J., Yan L., Yang Y., Zhao Z. (2021). Evaluation of the volatile profiles in pulp of 85 apple cultivars (*Malus domestica*) by HS-SPME combined with GC-MS. J. Food Meas. Charact..

[6] Li J., Lu T., Wang Y., Tang M., Ma T., Awasthi M.K., Sun X., Fang Y. (2025). Sensory-directed flavor decoding: Key aroma compounds determination in new Chinese apple cultivars using GC × GC-QTOFMS approach. Food Chem..

[7] Young H., Rossiter K., Wang M., Miller M. (1999). Characterization of Royal Gala Apple Aroma Using Electronic Nose Technology Potential Maturity Indicator. J. Agric. Food Chem..

[8] Zhao P., Yang Y., Wang X., Guo Y. (2021). Evolution of typical aromas and phenolic compounds of a red-fleshed apple throughout different fruit developmental periods in Xinjiang, China. Food Res. Int..

[9] Li K., Zhang D., Li Q., Chen J., Ge Y., Lv J., Li J., Mi H. (2026). Suppression of ethylene-responsive MdERF74/75 under low-oxygen storage disrupts aroma formation in apple fruit via fatty acid metabolism pathways. Postharvest Biol. Technol..

[10] Niu Y., Wang R., Xiao Z., Zhu J., Sun X., Wang P. (2019). Characterization of ester odorants of apple juice by gas chromatography-olfactometry, quantitative measurements, odour threshold, aroma intensity and electronic nose. Food Res. Int..

[11] Wang S., Chen H., Sun B. (2020). Recent progress in food flavor analysis using gas chromatography-ion mobility spectrometry (GC-IMS). Food Chem..

[12] Zhu W., Benkwitz F., Sarmadi B., Kilmartin P.A. (2021). Validation Study on the Simultaneous Quantitation of Multiple Wine Aroma Compounds with Static Headspace-Gas Chromatography-Ion Mobility Spectrometry. J. Agric. Food Chem..

[13] Yang Y., Zhu H., Chen J., Xie J., Shen S., Deng Y., Zhu J., Yuan H., Jiang Y. (2022). Characterization of the key aroma compounds in black teas with different aroma types by using gas chromatography electronic nose, gas chromatography-ion mobility spectrometry, and odor activity value analysis. LWT-Food Sci. Technol..

[14] Leng P., Hu H.W., Cui A.H., Tang H.J., Liu Y.G. (2021). HS-GC-IMS with PCA to analyze volatile flavor compounds of honey peach packaged with different preservation methods during storage. LWT-Food Sci. Technol..

[15] Chen Y., Li P., Liao L., Qin Y., Jiang L., Liu Y. (2021). Characteristic fingerprints and volatile flavor compound variations in Liuyang Douchi during fermentation via HS-GC-IMS and HS-SPME-GC-MS. Food Chem..

[16] Baltussen E., Sandra P., David F., Cramers C. (1999). Stir bar sorptive extraction (SBSE), a novel extraction technique for aqueous samples: Theory and principles. J. Microcolumn Sep..

[17] David F., Ochiai N., Sandra P. (2018). Two decades of stir bar sorptive extraction: A retrospective and future outlook. TrAC Trends Anal. Chem..

[18] Wang M., Ma W., Shi J., Zhu Y., Lin Z., Lv H. (2020). Characterization of the key aroma compounds in Longjing tea using stir bar sorptive extraction (SBSE) combined with gas chromatography-mass spectrometry (GC–MS), gas chromatography-olfactometry (GC-O), odor activity value (OAV), and aroma recombination. Food Res. Int..

[19] Cliff M., Stanich K., Trujillo J.M., Toivonen P., Forney C.F. (2011). Detemination and prediction of odor thresholds for odor active volatiles in a neutral apple juice matrix. Food Qual. Prefer..

[20] Van Gemert L.J. (2011). Odour Thresholds: Compilations of Odour Threshold Values in Air, Water and Other Media.

[21] Xiao N., Xu H., Jiang X., Sun T., Luo Y., Shi W. (2022). Evaluation of aroma characteristics in grass carp mince as affected by different washing processes using an E-nose, HS-SPME-GC-MS, HS-GC-IMS, and sensory analysis. Food Res. Int..

[22] Sun Z., Lin Y., Yang H., Zhao R., Zhu J., Wang F. (2024). Characterization of honey-like characteristic aroma compounds in Zunyi black tea and their molecular mechanisms of interaction with olfactory receptors using molecular docking. LWT-Food Sci. Technol..

[23] Yang S., Meng Z., Li Y., Chen R., Yang Y., Zhao Z. (2021). Evaluation of Physiological Characteristics, Soluble Sugars, Organic Acids and Volatile Compounds in ‘Orin’ Apples (*Malus domestica*) at Different Ripening Stages. Molecules.

[24] Li N., Li G., Li A., Tao Y. (2023). Synergy Effect between Fruity Esters and Potential Odorants on the Aroma of Hutai-8 Rose Wine Revealed by Threshold, S-Curve, and σ–τ Plot Methods. J. Agric. Food Chem..

[25] Pang X., Yin H., Li J., Shi Y., Yang Z. (2025). Molecular insights into the contribution of oak barrel aging to the aroma of beer with high alcohol content using SAFE-GC-O/AEDA and OAV calculation. Food Chem..

[26] Grosch W. (2001). Evaluation of the key odorants of foods by dilution experiments, aroma models and omission. Chem. Senses.

[27] Yan D., Shi J., Ren X., Tao Y., Ma F., Lia R., Liu X., Liu C. (2020). Insights into the aroma profiles and characteristic aroma of ‘Honeycrisp’ apple (Malus × domestica). Food Chem..

[28] Zhu D., Ren X., Wei L., Cao X., Ge Y., Liu H., Li J. (2020). Collaborative analysis on difference of apple fruits flavour using electronic nose and electronic tongue. Sci. Hortic..

[29] Donadel J.Z., Thewes F.R., Anese R.D., Schultz E.E., Berghetti M.R.P., Ludwig V., Klein B., Cichoski A.J., Barin J.S., Both V. (2019). Key volatile compounds of ‘Fuji Kiku’ apples as affected by the storage conditions and shelf life: Correlation between volatile emission by intact fruit and juice extracted from the fruit. Food Res. Int..

[30] Li R., Shi J., Li C., Ren X., Tao Y., Ma F., Liu Z., Liu C. (2023). Characterization of the key odorant compounds in ‘Qinguan’ apples (Malus × domestica). LWT-Food Sci. Technol..

[31] Mehinagic E., Royer G., Symoneaux R., Jourjon F., Prost C. (2006). Characterization of odor-active volatiles in apples: Influence of cultivars and maturity stage. J. Agric. Food Chem..

[32] Defilipi B.G., Kader A.A., Dandekar A.M. (2005). Apple aroma: Alcohol acyltransferase, a rate-limiting step for ester biosynthesis, is regulated by ethylene. Plant Sci..

[33] Song S., Zhang X., Hayat K., Huang M., Liu P., Karangwa E., Gu F., Jia C., Xia S., Xiao Z. (2010). Contribution of beef base to aroma characteristics of beeflike process flavour assessed by descriptive sensory analysis and gas chromatography olfactometry and partial least squares regression. J. Chromatogr. A.

[34] Holland D., Larkov O., Bar-Ya’akov I., Bar E., Zax A., Brandeis E., Ravid U., Lewinsohn E. (2005). Developmental and Varietal Differences in Volatile Ester Formation and Acetyl-CoA:  Alcohol Acetyl Transferase Activities in Apple (*Malus domestica Borkh*.) Fruit. J. Agric. Food Chem..

[35] Peng B., Yu M., Zhang B., Xu J., Ma R. (2020). Differences in PpAAT1 activity in high- and low-aroma peach varieties affect γ-decalactone production. Plant Physiol..

[36] Shen D., Song H., Zou T., Wan S., Li M. (2022). Characterization of odor-active compounds in moso bamboo (*Phyllostachys pubescens* Mazel) leaf via gas chromatography-ion mobility spectrometry, one- and two-dimensional gas chromatography-olfactory-mass spectrometry, and electronic nose. Food Res. Int..

[37] Duensing P.W., Hinrichs J., Schieberle P. (2024). Influence of Milk pasteurization on the key aroma compounds in a 30 weeks ripened pilot-scale gouda cheese elucidated by the Sensomics approach. J. Agric. Food Chem..

[38] Niu Y., Zhang X., Zhang X., Xiao Z., Song S., Eric K., Jia C., Yu H., Zhu J. (2011). Characterization of odor-active compounds of various cherry wines by gas chromatography-mass spectrometry, gas chromatography-olfactometry and their correlation with sensory attributes. J. Chromatogr. B.

